# Supplementary material for: Remote and precise control over morphology and motion of organic crystals by using magnetic field
Source: Nat Commun. 2022 Apr 28;13:2322. doi: 10.1038/s41467-022-29959-1 (PMC9050695; doi:10.1038/s41467-022-29959-1)
Supplement: Supplementary file 1 — Supplementary Information [file 41467_2022_29959_MOESM1_ESM.pdf]

Supplementary Information

Remote and Precise Control over Morphology and Motion of Organic Crystals by using Magnetic Field

Xuesong Yang[1], Linfeng Lan,[1] Liang Li,[2,3] Xiaokong Liu,[1] Pančе Naumov,[2,4]* and Hongyu Zhang[1]*

[1]*State Key Laboratory of Supramolecular Structure and Materials, College of Chemistry, Jilin University, Changchun 130012, P. R. China*

[2]*Smart Materials Lab, New York University Abu Dhabi, PO Box 129188, Abu Dhabi, UAE*

[3]*Department of Sciences and Engineering, Sorbonne University Abu Dhabi, PO Box 38044, Abu Dhabi, UAE*

[4]*Molecular Design Institute, Department of Chemistry, New York University, 100 Washington Square East, New York, NY 10003, USA*

*Corresponding authors. Emails for correspondence: pance.naumov@nyu.edu; hongyuzhang@jlu.edu.cn

Supplementary Methods

General information

The ^{1}H and $^{13}C\{^{1}H\}$ NMR spectra were recorded on Agar Scientifica 400 MHz or Bruker Avance 500 MHz spectrometers with tetramethylsilane as internal standard. The mass spectra were recorded on Thermo Fisher ITQ1100 mass spectrometer. Elemental analyses were performed on Elementar Vario Micro Cube analyzer. The scanning electron microscopy (SEM) images were recorded using FEI Quanta 450 operated at 5–10 kV. The emission spectra were recorded on Maya2000 Pro CCD spectrometer. To study the optical waveguiding properties, the crystals were irradiated by the third harmonic (355 nm) of a Nd:YAG (yttrium-aluminum-garnet) laser at a repetition rate of 10 Hz and pulse duration of about 10 ns. The energy of the laser was adjusted by using calibrated neutral density filters. The beam was focused on a stripe whose shape was adjusted to 3.3 × 0.6 mm by using cylindrical lens and a slit. The edge emission spectra were recorded on Maya2000 Pro CCD spectrometer. The magnetic field (B) was measured by using a SA-3-A Gaussmeter and a magnet (diameter 36 mm, thickness 7 mm, vertical pull 6–29 kg).

Crystallization procedure

Dichloromethane solutions of compounds **1**, **2**, **3**, **4**, **5** and **6** were added to test tubes, and then an antisolvent was carefully layered on top of the solutions for slow diffusion. The antisolvent for compounds **1**, **3**, **4** and **5** was a triple volume of ethanol, in case of compound **2** it was a double volume of petroleum ether, and for compound **6** it was a triple volume of cyclohexane. Needle-like crystals of **1**−**6** were obtained after one to two weeks at room temperature.

Synthetic procedures

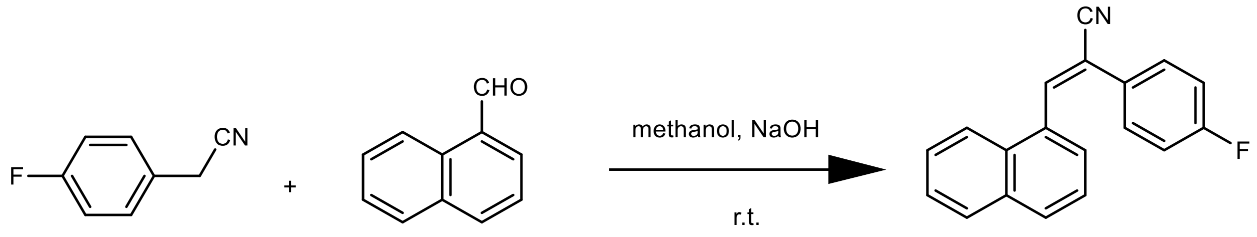

1

Supplementary Figure 1. Synthetic procedure for compound 1.

4-Fluorophenylacetonitrile (1.36 g, 10 mmol) and 1-naphthaldehyde (1.56 g, 10 mmol) were added to methanol (50 mL). NaOH (0.40 g, 10 mmol) was added, and the mixture was stirred for 1 h at room temperature. The reaction mixture was filtered to give a green solid, which was dissolved in dichloromethane and washed with brine. After drying over Na_2SO_4, the solvent was removed by vacuum evaporation. The resulting crude product was purified by column chromatography using dichloromethane as eluent to obtain compound **1** (2.50 g, 86%) as a green powder. ^{1}H NMR (Chloroform-*d*, 400 MHz) δ 8.22 (1H, s), 8.07 (1H, d, J = 7.2 Hz), 8.01 – 7.88 (3H, m), 7.80 – 7.71 (2H, m), 7.63 – 7.52 (3H, m), 7.19 (2H, t, J = 8.5 Hz). ^{13}C NMR (Chloroform-*d*, 126 MHz) δ 164.37, 162.38, 140.17, 133.54, 131.51, 131.00, 130.87, 130.39, 129.02, 128.15, 128.08, 127.04, 126.49, 125.55, 123.30, 117.59, 116.35, 116.17, 114.26. MS (ESI+): m/z calcd for $C_{19}H_{12}FN$ $[M+H]^+$: 273.10; Found: 273.14. Anal. calcd (%) for $C_{19}H_{12}FN$: C, 83.50; H, 4.43; N, 5.12. Found: C, 84.06; H, 4.55; N, 5.15.

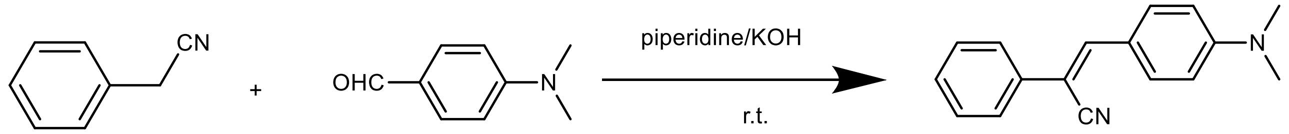

2

Supplementary Figure 2. Synthetic procedure for compound 2.

2-Phenylacetonitrile (1.17 g, 10 mmol) and 4-(dimethylamino)benzaldehyde (1.49 g, 10 mmol) were added to ethanol (50 mL). Piperidine (0.30 mL) was then added, and the mixture was stirred for 1 h at room temperature. The reaction mixture was filtered to give a green solid, which was dissolved in dichloromethane and washed with brine. After drying over Na_2SO_4, the solvent was removed by vacuum evaporation. The resulting crude product was purified by column chromatography using dichloromethane as eluent to obtain compound **2** (2.10 g, 79%) as a green powder. ^{1}H NMR (Chloroform-*d*, 500 MHz) δ 7.92 – 7.77 (2H, m), 7.62 (2H, d, J = 7.7 Hz), 7.41 – 7.29 (4H, m), 6.70 (2H, d, J = 8.8 Hz), 3.03 (6H, s). ^{13}C NMR (Chloroform-*d*, 126 MHz) δ 151.67, 142.58, 135.60, 131.32, 129.42, 128.99, 125.48, 121.65, 119.50, 111.66, 104.53, 40.99. MS (ESI+): m/z calcd for $C_{17}H_{16}N_2$ $[M+H]^+$: 248.13; Found: 248.18. Anal. calcd (%) for $C_{17}H_{16}N_2$: C, 80.22; H, 6.49; N, 11.28. Found: C, 80.54; H, 6.34; N, 11.40.

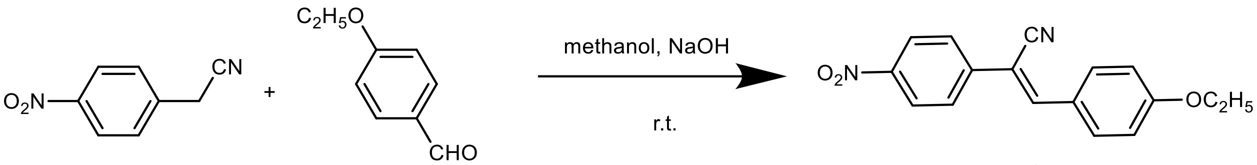

3

Supplementary Figure 3. Synthetic procedure for compound 3.

4-Nitrophenylacetonitrile (1.62 g, 10 mmol) and 4-ethoxybenzaldehyde (1.51 g, 10 mmol) were added to methanol (50 mL). NaOH (0.40 g, 10 mmol) was added, and the mixture was stirred for 1 h at room temperature. The reaction mixture was filtered to give a yellow solid, which was dissolved in dichloromethane and washed with brine. After drying over Na_2SO_4, the solvent was removed by vacuum evaporation. The resulting crude product was purified by column chromatography using dichloromethane as eluent to obtain compound **3** (2.80 g, 89%) as a yellow powder. ^{1}H NMR (DMSO-d_6, 500 MHz) δ 8.38 – 8.32 (2H, m), 8.23 (1H, s), 8.06 – 7.98 (4H, m), 7.17 – 7.08 (2H, m), 4.15 (2H, q, J = 7.0 Hz), 1.37 (3H, t, J = 7.0 Hz). ^{13}C NMR (DMSO-d_6, 126 MHz) δ 161.75, 147.42, 146.51, 132.38, 126.99, 126.09, 124.78, 118.35, 115.54, 105.21, 64.07, 14.97. MS (ESI+): m/z calcd for $C_{19}H_{12}N_2O_3$ $[M+H]^+$: 294.10; Found: 294.11. Anal. calcd (%) for $C_{19}H_{12}N_2O_3$: C, 69.38; H, 4.79; N, 9.52. Found: C, 69.90; H, 4.95; N, 9.55. Note: minor presence of geometrical isomers in compound **3** will not affect the elemental analysis results since both isomers have the same molecular formula.

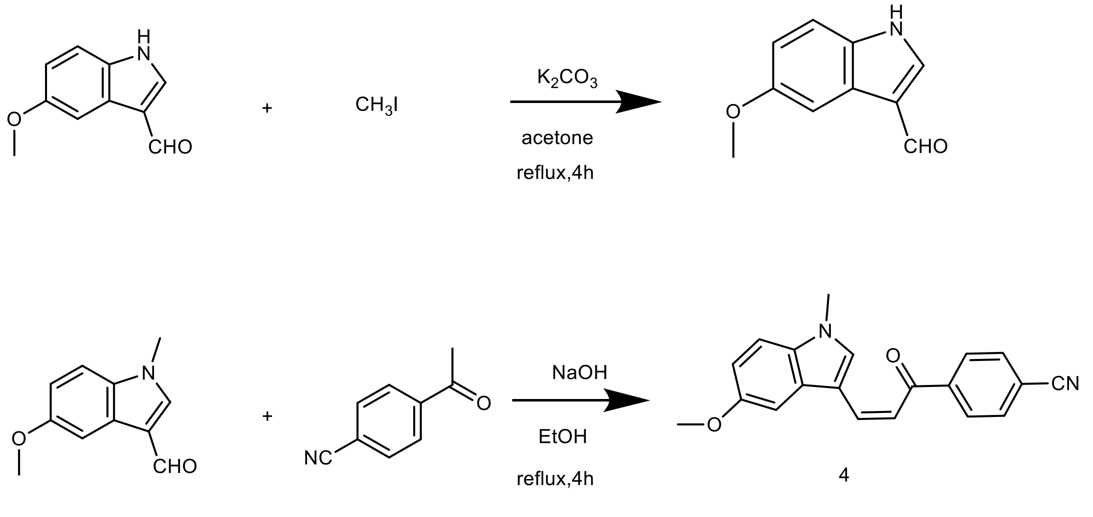

Supplementary Figure 4. The synthetic procedure for compound 4.

5-Methoxy-1H-indole-3-carbaldehyde (1.75 g, 10 mmol) was dissolved in 50 mL acetone, and K_2CO_3 (4.15 g, 30 mmol) was added. Then 1 mL CH_3I was injected into the solvent, and the mixture was refluxed for 4 h. After cooling to room temperature, the K_2CO_3 was filtered out, and the solvent was removed by distillation under reduced pressure. 80 mL ethanol, 4-acetylbenzonitrile (1.45 g, 10 mmol) and NaOH (0.08 g, 2 mmol) were added to the residue, and the mixture was refluxed for 4 h. The obtained precipitate was filtered and washed with ethanol to give the crude product, which was purified by vacuum sublimation to produce compound **4** as a yellow solid (1.99 g, 63% yield). ^{1}H NMR (DMSO-d_6, 500 MHz) δ 8.24 (1H, d, J = 1.7 Hz), 8.22 (1H, d, J = 2.0 Hz), 8.13 (1H, s), 8.04 (2H, d, J = 3.7 Hz), 8.02 (1H, d, J = 1.8 Hz), 7.56 (1H, s), 7.50 (1H, d, J = 2.4 Hz), 7.47 (1H, s),

6.97 (1H, d, J = 2.4 Hz), 3.88 (3H, s), 3.84 (3H, s). ^{13}C NMR (DMSO-d_6, 126 MHz) δ 188.18, 155.89, 142.61, 140.21, 137.47, 133.52, 133.16 (2C) , 129.15 (2C), 127.02, 118.88, 114.82, 114.67, 112.64, 112.21, 112.04, 103.40, 56.11, 33.77. MS (ESI+): m/z calcd for $C_{20}H_{16}N_2O_2$ $[M+H]^+$: 316.12; Found: 316.33. Anal. calcd (%) for $C_{20}H_{16}N_2O_2$: C, 75.93; H, 5.10; N, 8.86. Found: C, 76.16; H, 5.15; N, 8.73.

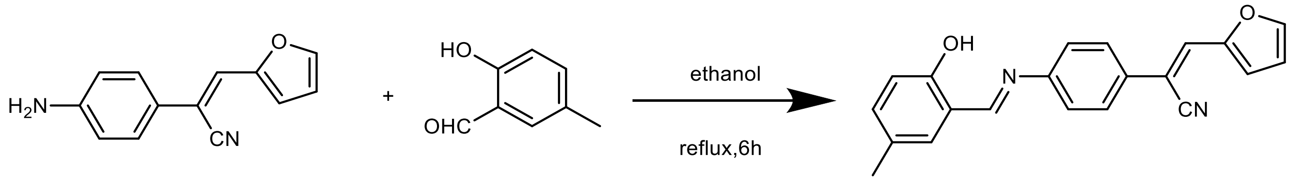

5

Supplementary Figure 5. Synthetic procedure for compound 5.

2-(4-aminophenyl)-3-(furan-2-yl)acrylonitrile (2.10 g, 10 mmol) and 2-hydroxy-5-methylbenzaldehyde (1.36 g, 10 mmol) were dissolved in ethanol (50 mL). After refluxing for 6 h, the resulting mixture was cooled down to room temperature, filtered and washed with ethanol. The crude product was purified by column chromatography using dichloromethane and petroleum ether (V/V = 4:1) as the eluent to produce compound **5** as orange-red solid (3.02 g, 92.1% yield). ^{1}H NMR (Chloroform-d, 400 MHz) δ 8.61 (1H, s), 7.71 (2H, dd, J = 8.5, 1.4 Hz), 7.62 (1H, d, J = 1.7 Hz), 7.41 (1H, s), 7.38 – 7.29 (2H, m), 7.25 – 7.17 (3H, m), 6.95 (1H, d, J = 8.9 Hz), 6.60 (1H, dt, J = 3.4, 1.5 Hz), 2.33 (3H, s). ^{13}C NMR (DMSO-d_6, 126 MHz) δ 164.04, 158.63, 150.20, 149.10, 146.83, 134.80, 132.73, 132.15, 128.50, 128.25, 126.96, 122.67, 119.48, 118.01, 117.59, 116.97, 113.69, 105.57, 20.40. MS (ESI+): m/z calcd for $C_{21}H_{16}N_2O_2$ $[M+H]^+$: 328.12; Found: 328.06. Anal. calcd (%) for $C_{21}H_{16}N_2O_2$: C, 76.81; H, 4.91; N, 8.53. Found: C, 76.97; H, 4.99; N, 8.68.

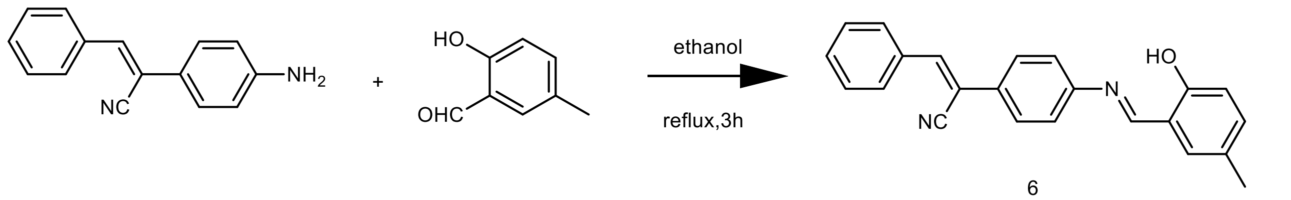

6

Supplementary Figure 6. Synthetic procedure for compound 6.

2-(4-aminophenyl)-3-phenylacrylonitrile (2.20 g, 10 mmol) and 2-hydroxy-5-methylbenzaldehyde (1.36 g, 10 mmol) were heated in ethanol (120 mL) at 80 °C for 3 h. After cooling to room temperature, the mixture was filtered. The filter cake was dried and purified by silica gel column chromatography using dichloromethane as eluent to produce compound **6** as an orange solid (3.04 g, 90.2% yield). ^{1}H NMR (Methylene Chloride-d_2, 500 MHz) δ 12.75 (1H, s), 8.66 (1H, s), 7.91 (2H, d, J = 7.3 Hz), 7.77 (2H, d, J = 7.7 Hz), 7.62 (1H, s), 7.50 (3H, t, J = 7.8 Hz), 7.39 (2H, d, J = 7.7 Hz), 7.28 – 7.19 (2H, m), 6.91 (1H, d, J = 8.3 Hz), 2.33 (3H, s). ^{13}C NMR (Chloroform-d, 126 MHz) δ 163.22, 159.05, 149.33, 141.88, 134.55, 133.67, 132.86, 132.45, 130.64, 129.31, 129.01, 128.35, 127.08, 121.88, 118.73, 117.88, 117.12, 110.96, 20.36. MS (ESI+): m/z calcd for $C_{23}H_{18}N_2O$ $[M+H]^+$: 338.14, Found: 338.07. Anal. calcd (%) for $C_{23}H_{18}N_2O$: C, 81.63; H, 5.36; N, 8.28. Found: C, 81.61; H, 5.33; N, 8.29.

Supplementary Figures

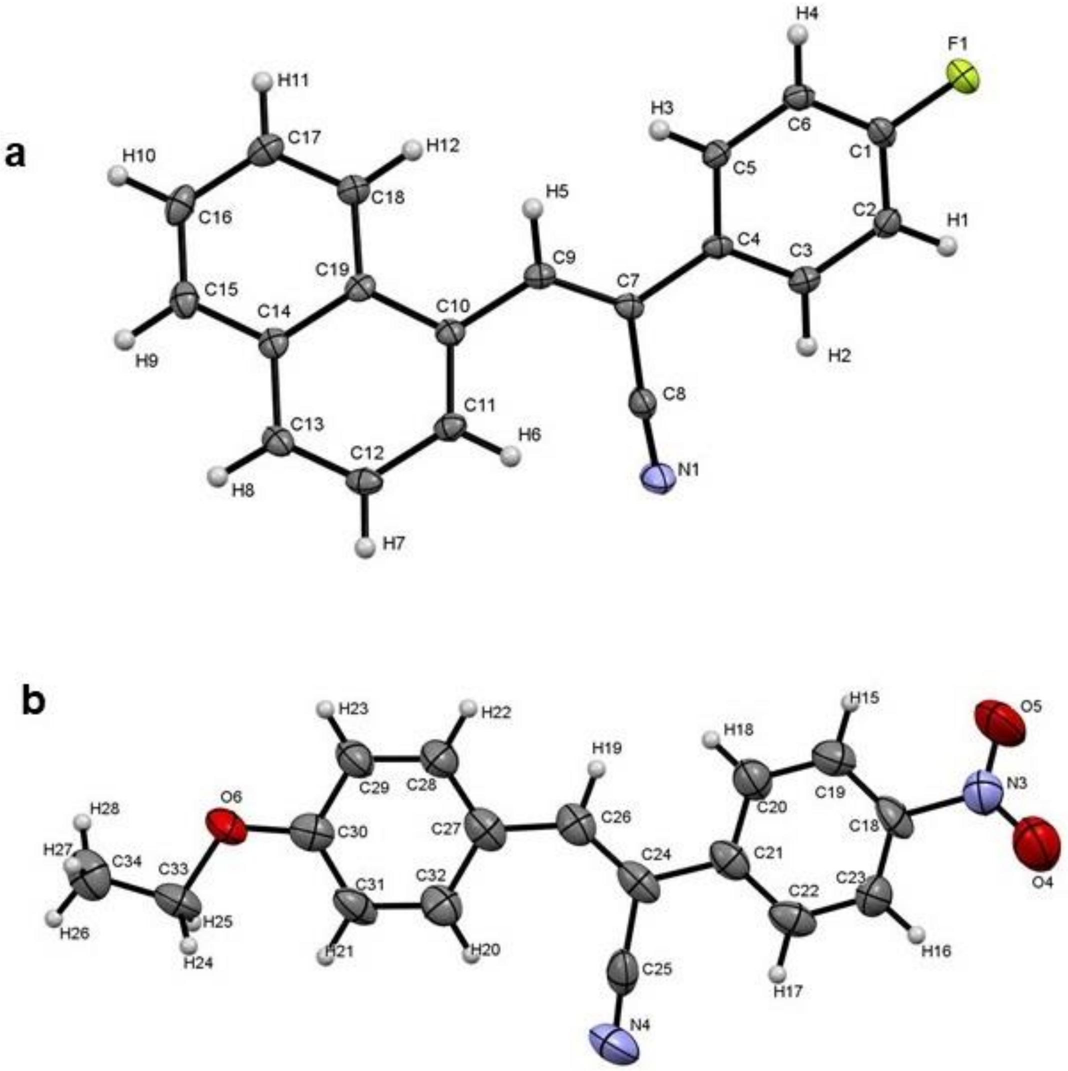

Supplementary Figure 7. ORTEP-style plot of the molecular structures of compounds 1 (a) and 3 (b) shown at 50% probability.

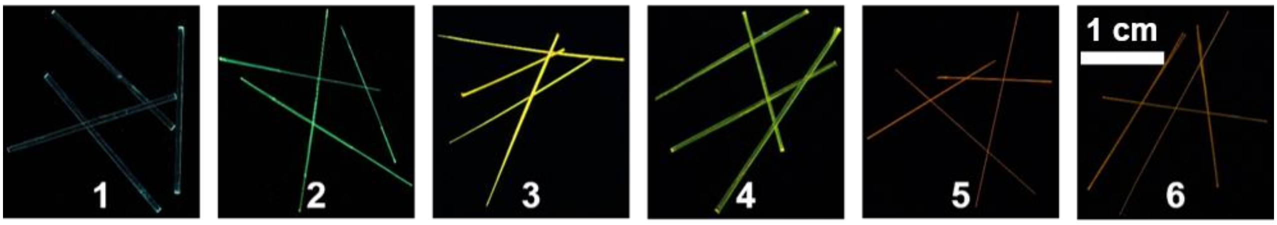

Supplementary Figure 8. Photographs of crystals of 1−6 under 365 nm radiation.

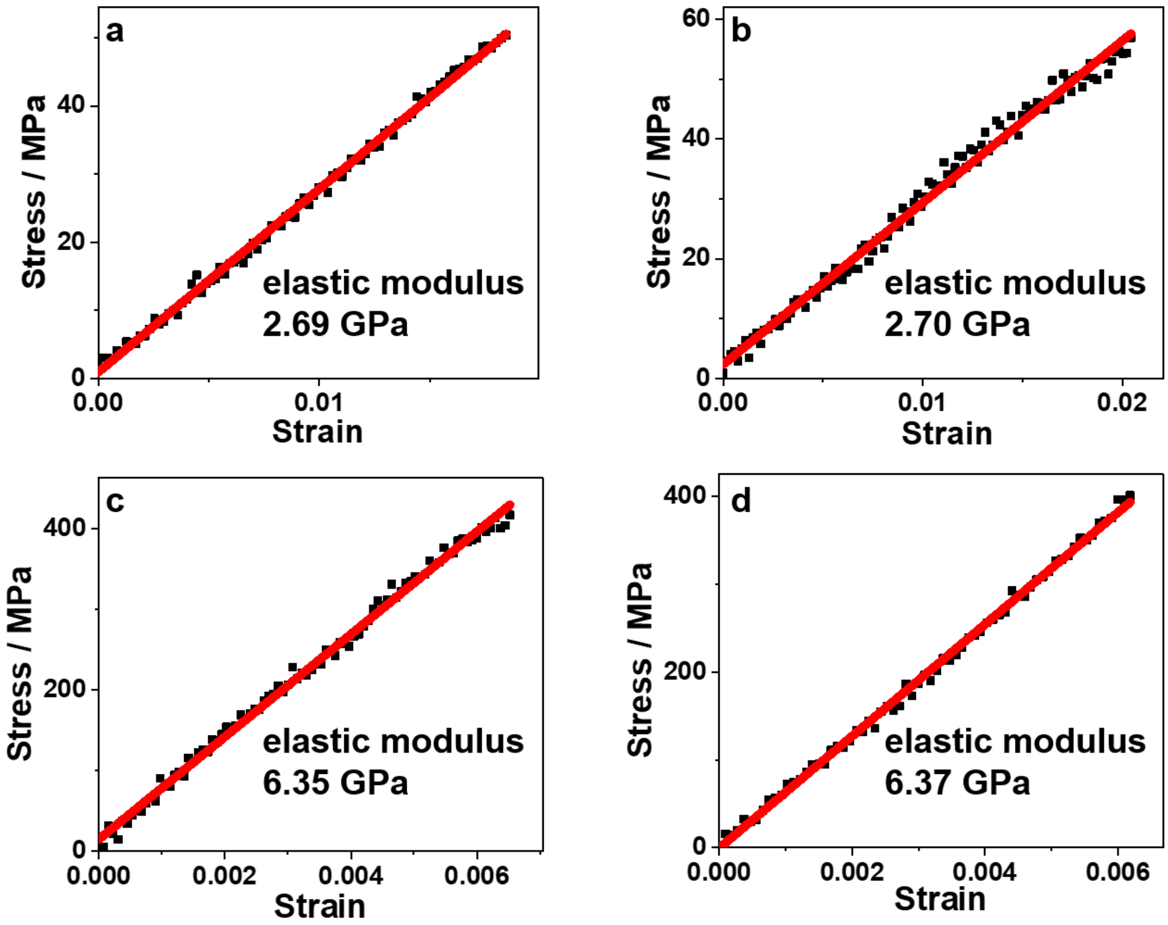

Supplementary Figure 9. Mechanical properties of the crystals. (a) **1**, (b) MNP//**1**, (c) **3**, (d) MNP//**3**.

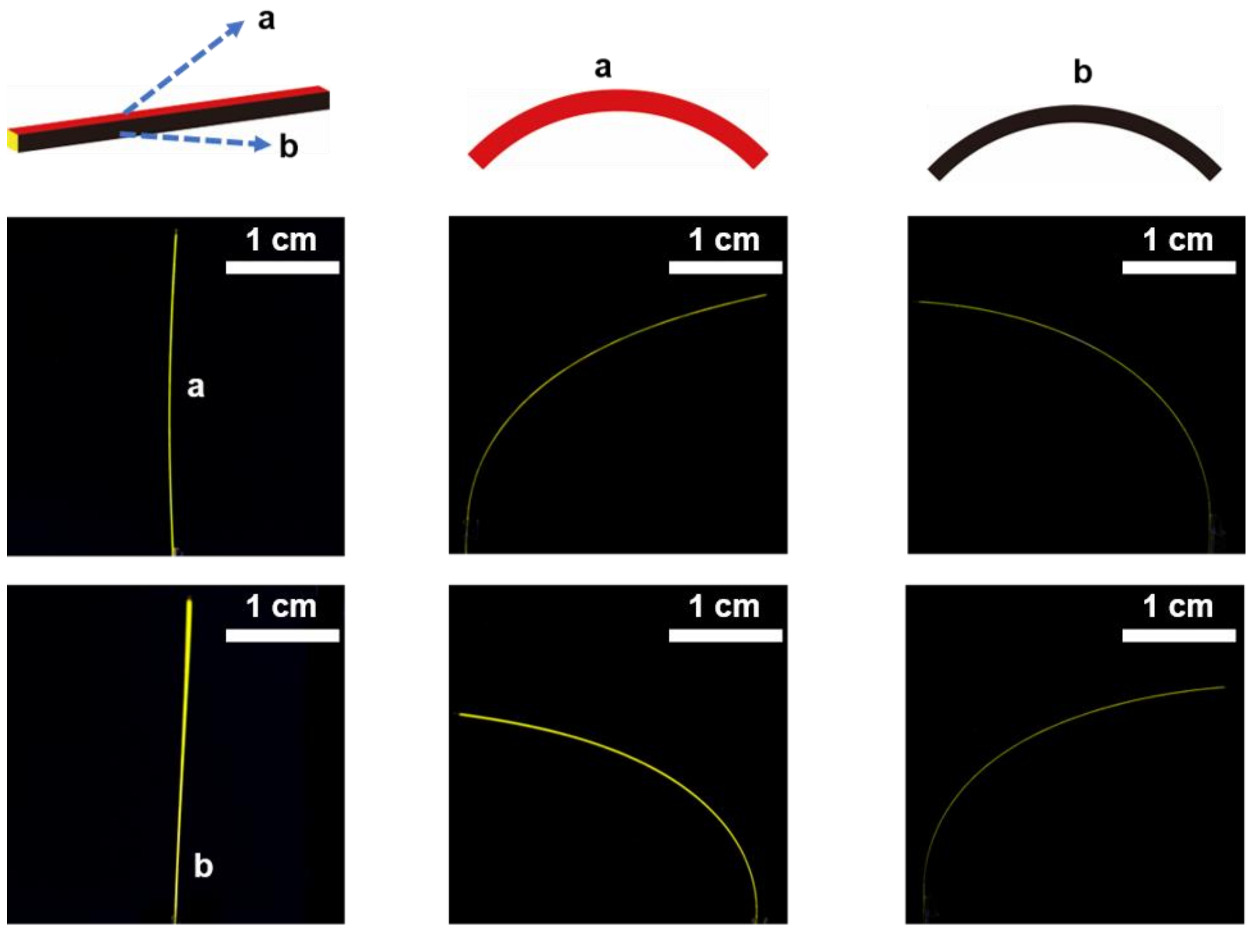

Supplementary Figure 10. Photographs of bending of crystal 4 on two different faces.

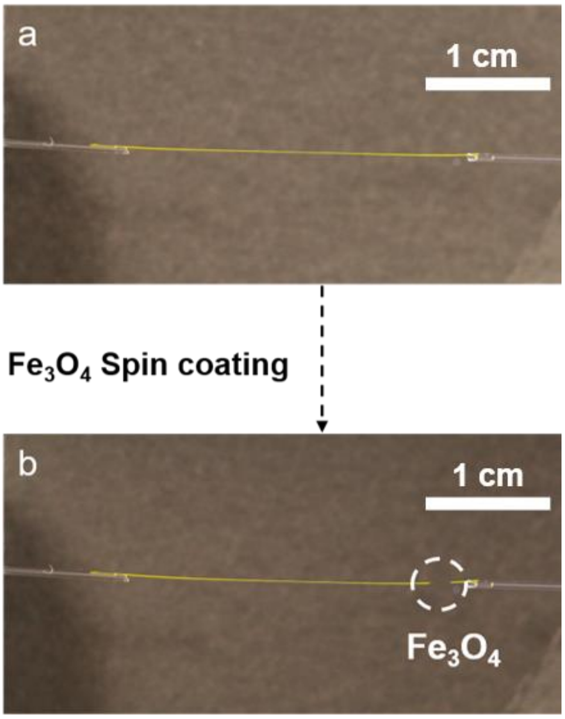

Supplementary Figure 11. Preparation of the hybrid crystals. (a) PDDA/PSS//**4**. (b) MNP//**4**.

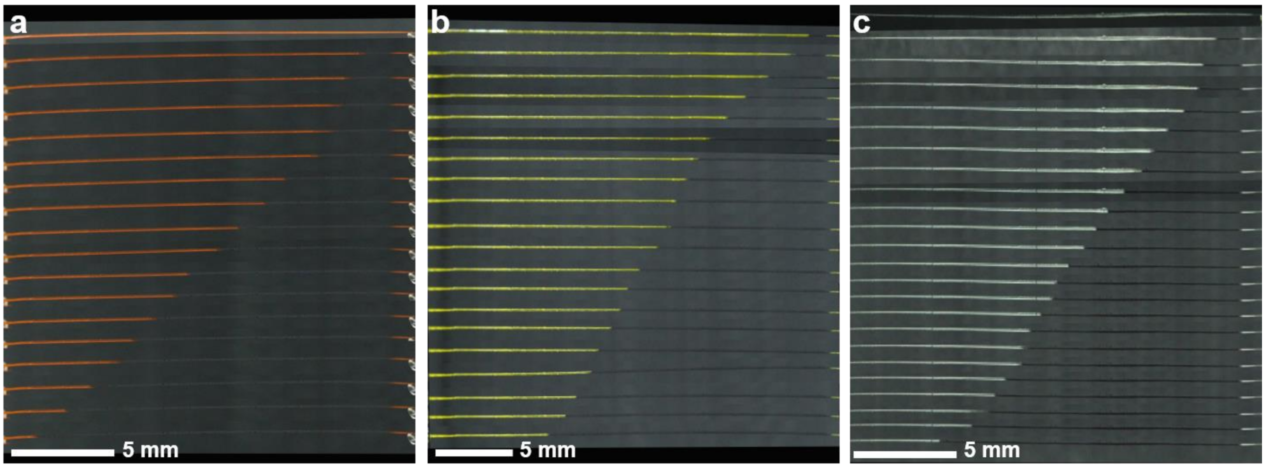

Supplementary Figure 12. Photographs of different lengths of Fe_3O_4 MNPs coatings assembled on the crystals. (a) MNP//**1**, (b) MNP//**4**, (c) MNP//**5**.

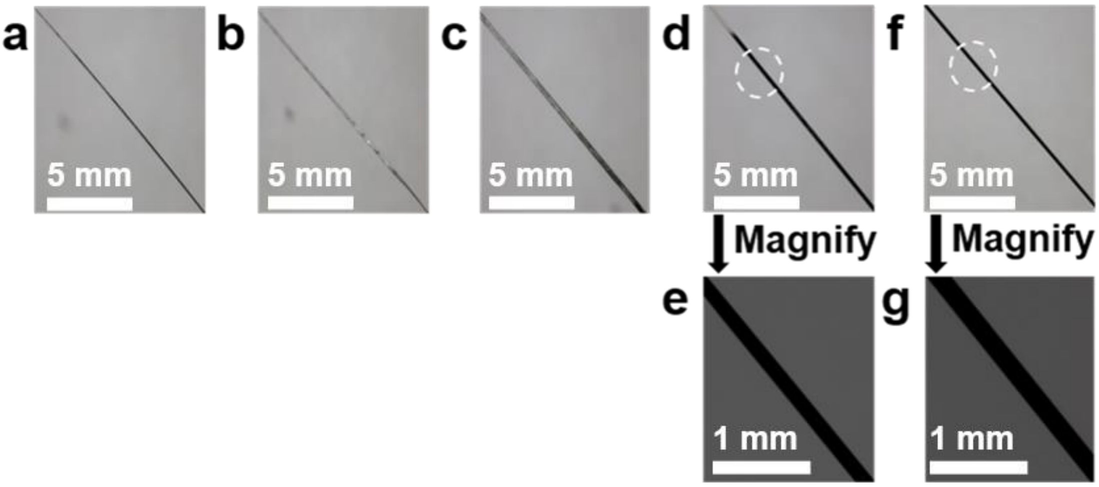

Supplementary Figure 13. Photographs of a crystal of PDDA/PSS//1 after application of aqueous suspensions of iron(III) oxide with varying concentrations. (a) 0.25 g/mL, (b) 0.5 g/mL, (c)1.0 g/mL, (d,e) 2.0 g/mL, (f,g) 3.0 g/mL.

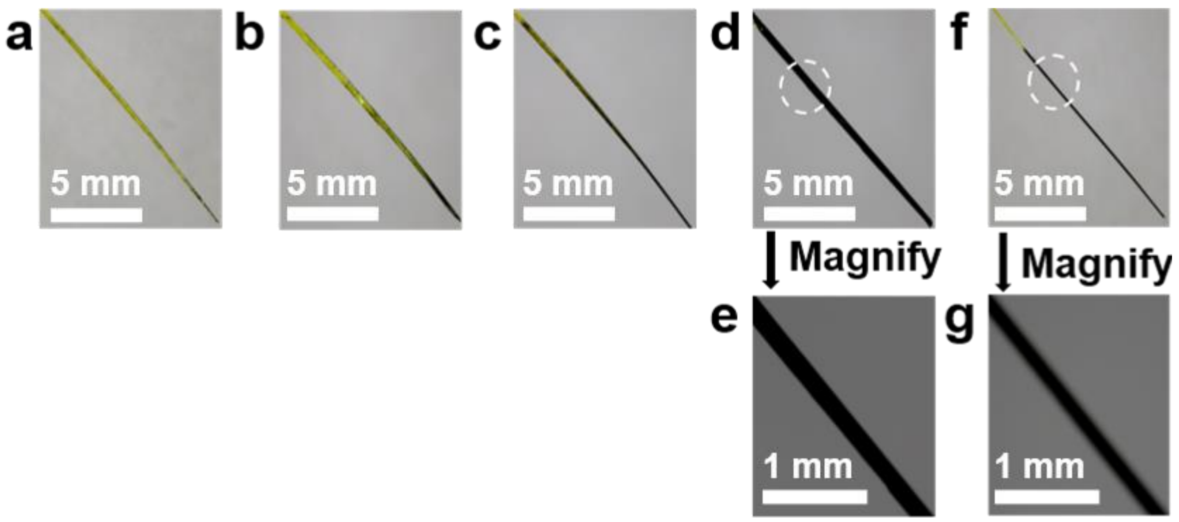

Supplementary Figure 14. Photographs of a crystal of PDDA/PSS//3 after application of aqueous suspensions of iron(III) oxide with varying concentrations. (a) 0.25 g/mL, (b) 0.5 g/mL, (c)1.0 g/mL, (d,e) 2.0 g/mL, (f,g) 3.0 g/mL.

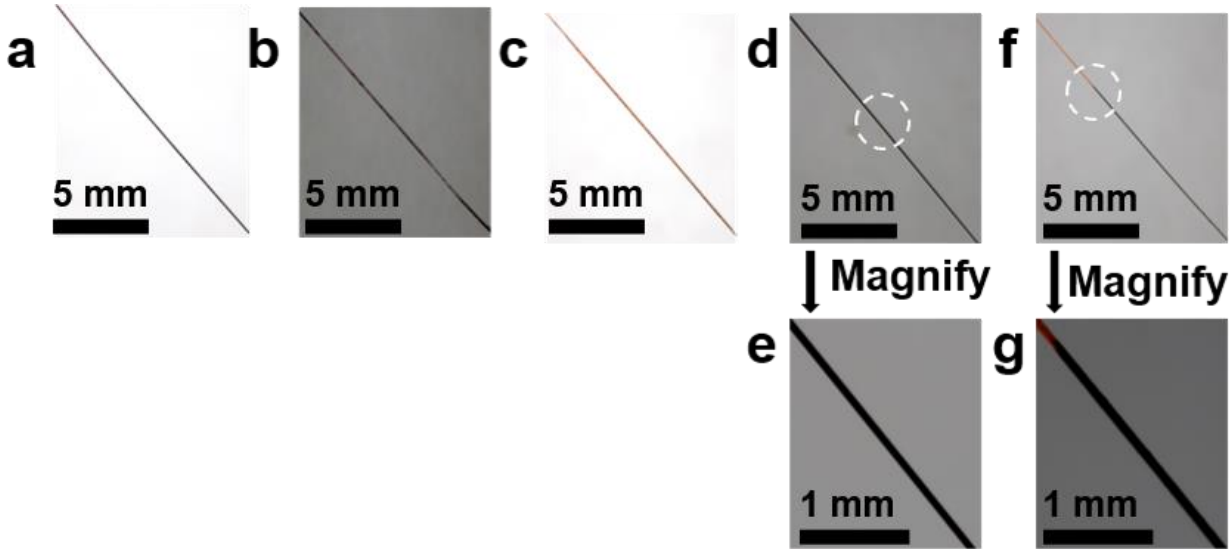

Supplementary Figure 15. Photographs of a crystal of PDDA/PSS//5 after application of aqueous suspensions of iron(III) oxide with varying concentrations. (a) 0.25 g/mL, (b) 0.5 g/mL, (c)1.0 g/mL, (d,e) 2.0 g/mL, (f,g) 3.0 g/mL.

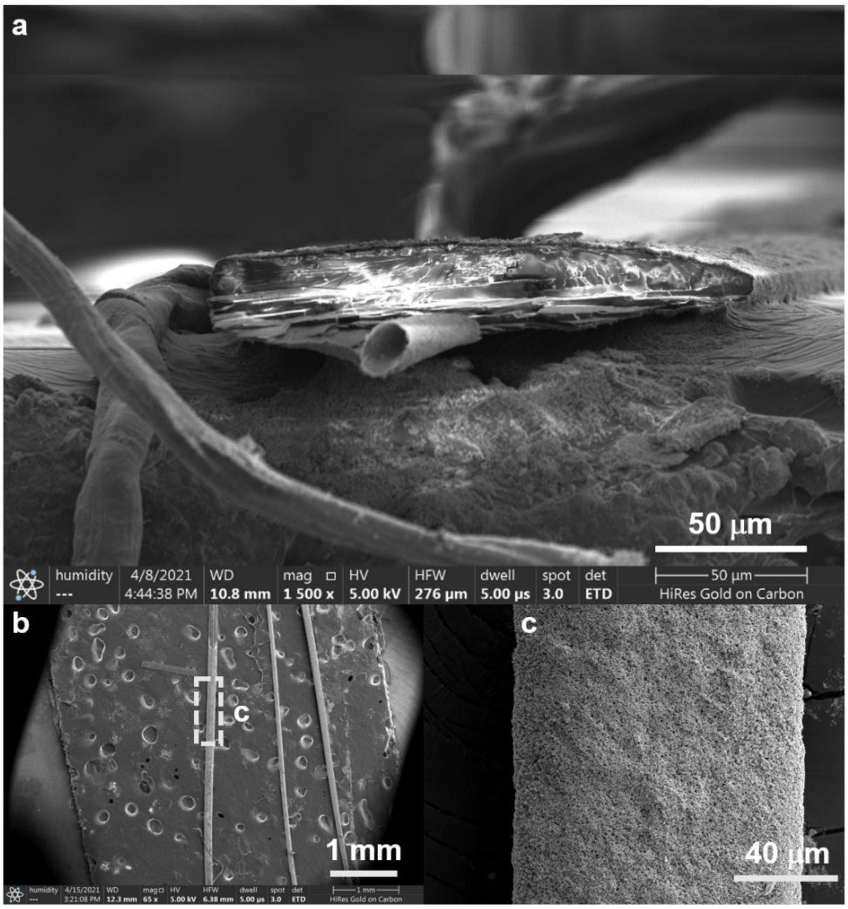

Supplementary Figure 16. **Scanning electron micrographs of a crystal of MNP//1.** (a) Cross-section of the crystal. (b,c) Surface of the crystal.

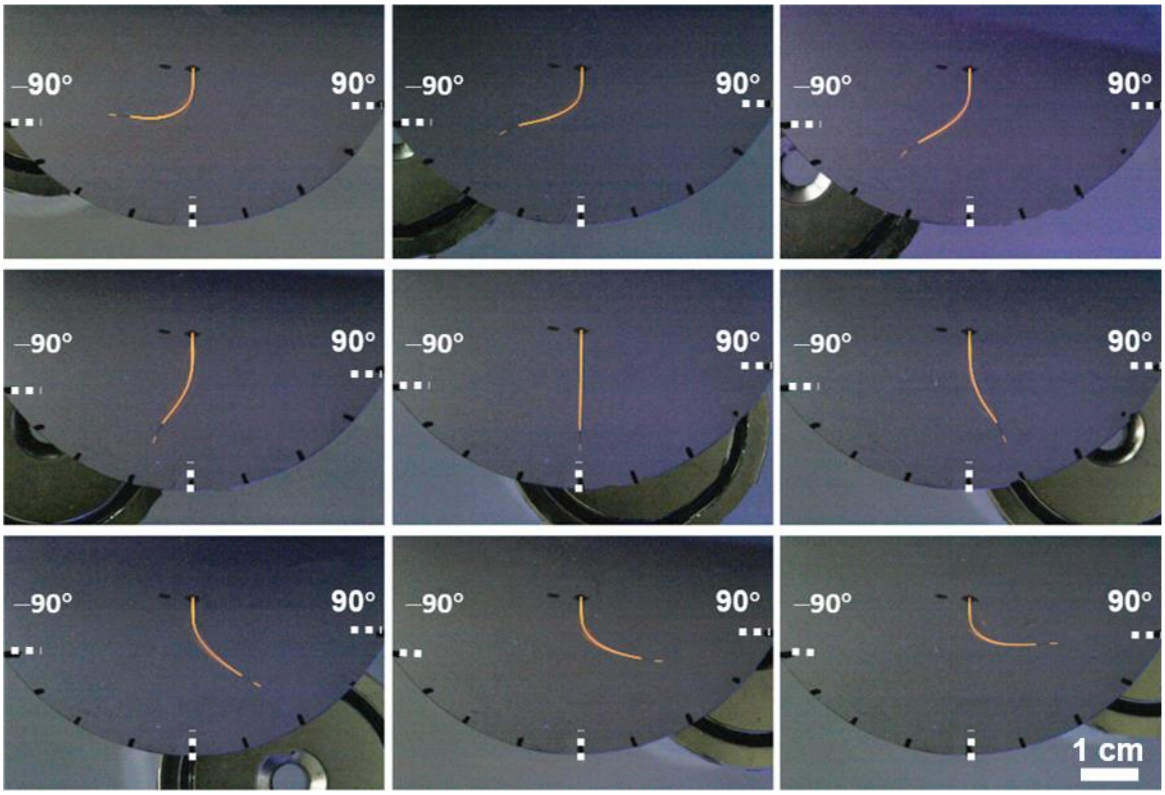

Supplementary Figure 17. Photographs of a crystal of MNP//6 bending precisely towards 9 points on a silicon wafer. Note that the contrast of color in these images was enhanced for clarity.

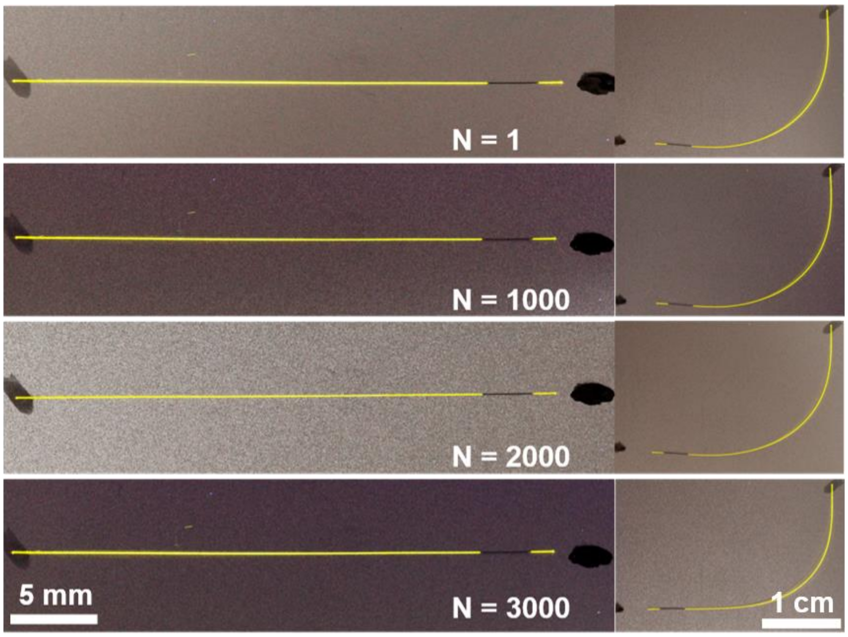

Supplementary Figure 18. MNP//4 durability test at 50 °C. *N* is the number of bending cycles.

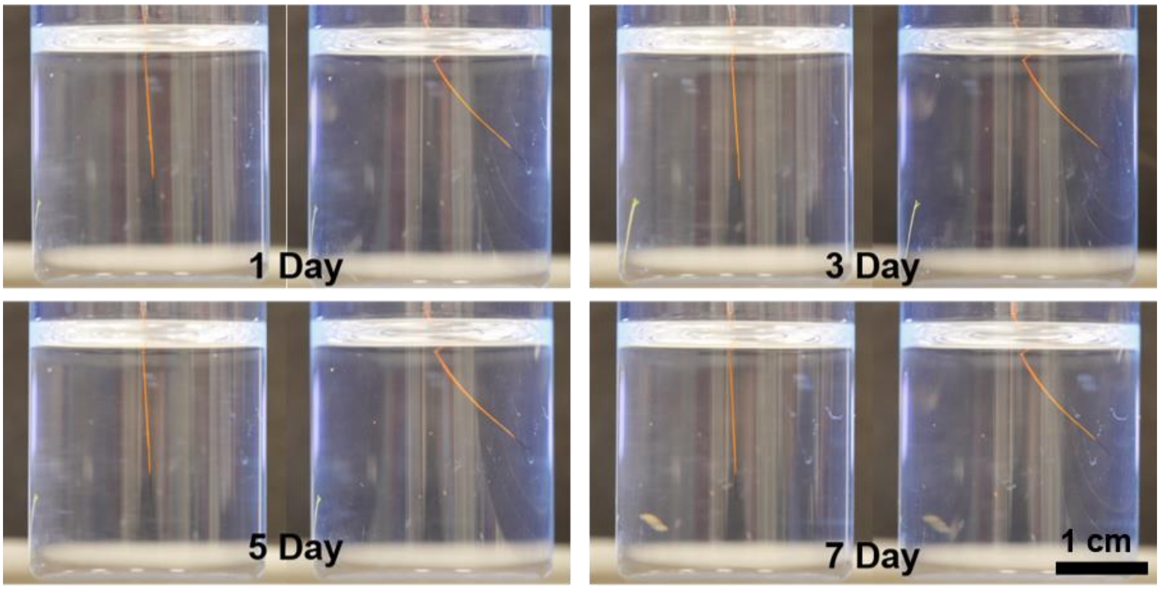

Supplementary Figure 19. Photograph of MNP//5 placed in water over different periods of time.

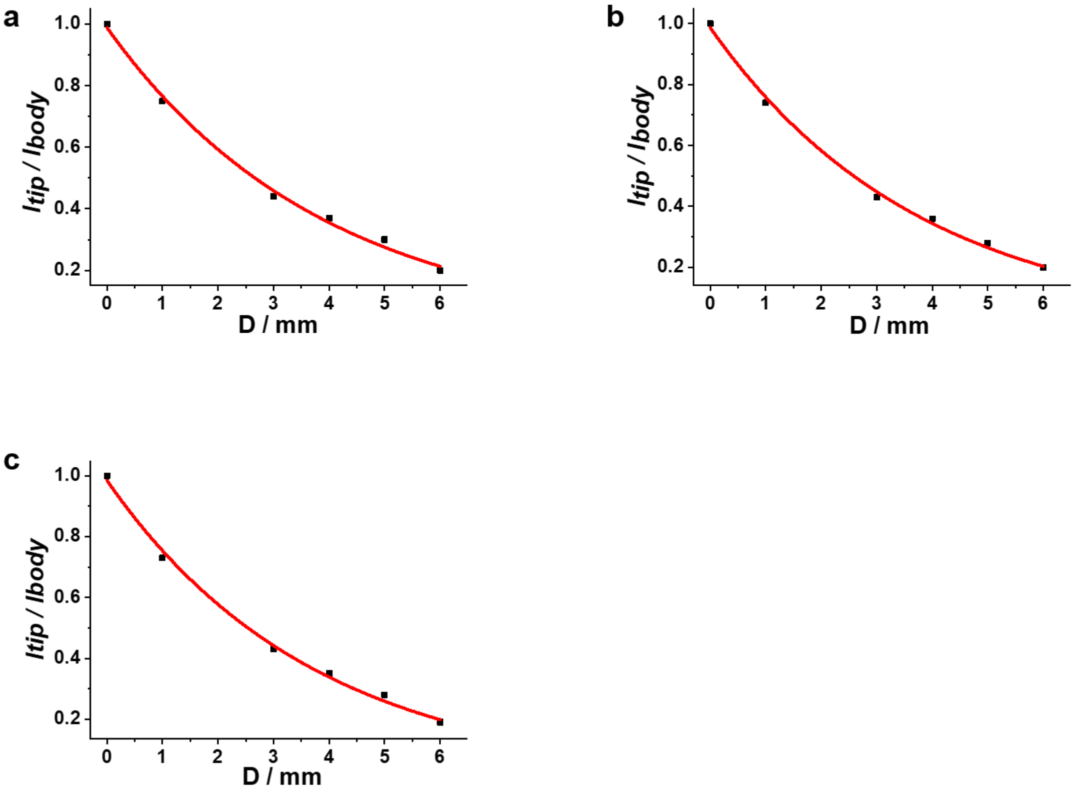

Supplementary Figure 20. Decay of light intensity with distance, I_{tip}/I_{body}. (a) uncoated straight crystal, **4**. (b) coated straight crystal, MNP//**4**, (c) and coated bent crystal.

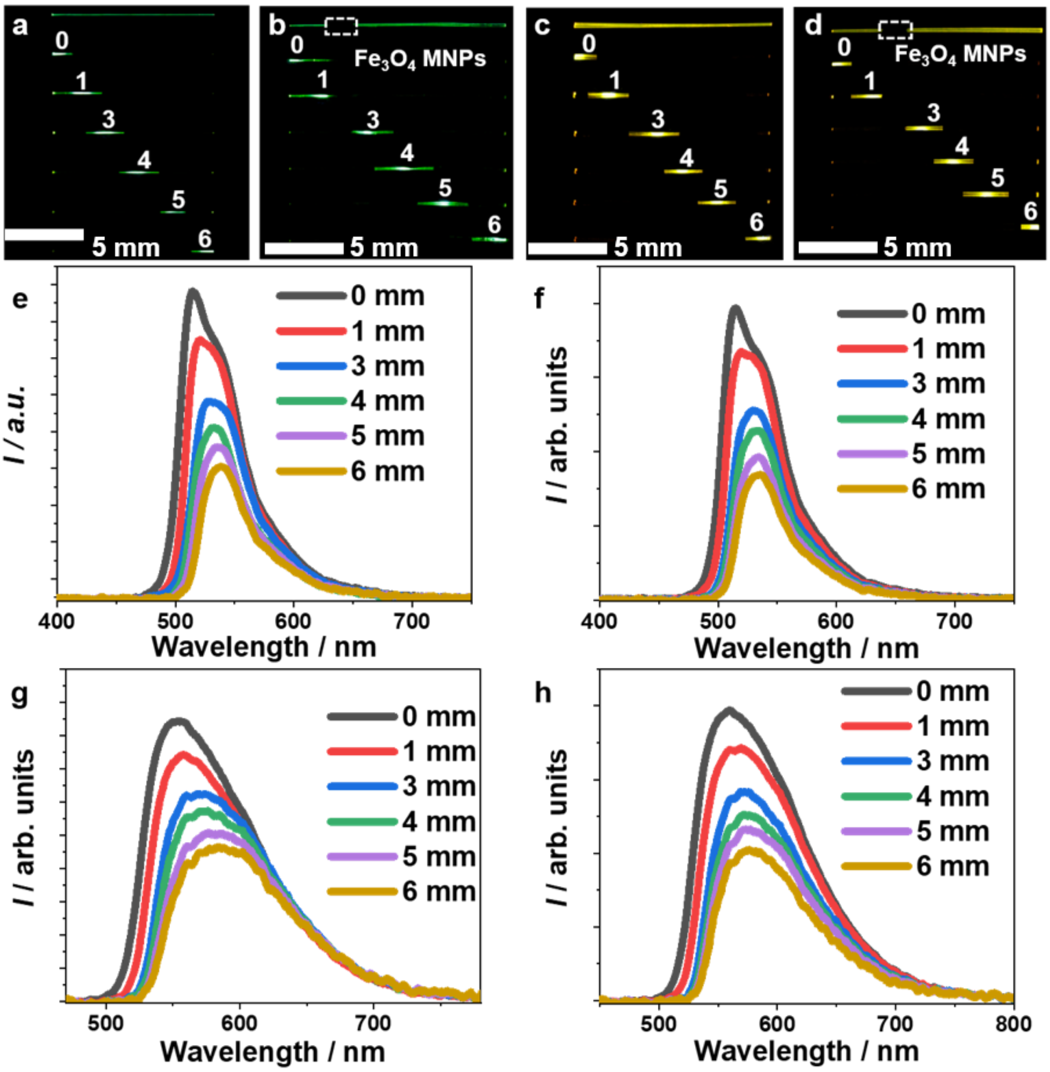

Supplementary Figure 21. Optical waveguiding properties. (a,b) Images of a crystal of **2** used as a waveguide in uncoated straight state, **2** (a), and coated straight state, MNP//**2**, (b). (c,d) Images of a crystal of **3** used as a waveguide in uncoated straight state, **3** (c), and coated straight state, MNP//**3**, (d). (e–h) Fluorescence spectra collected at one tip of the crystal with different distances between the tip and the excitation site of the 355 nm light. The spectra in panels e, f, g and h correspond to the crystals shown in panels a, b, c and d, respectively.

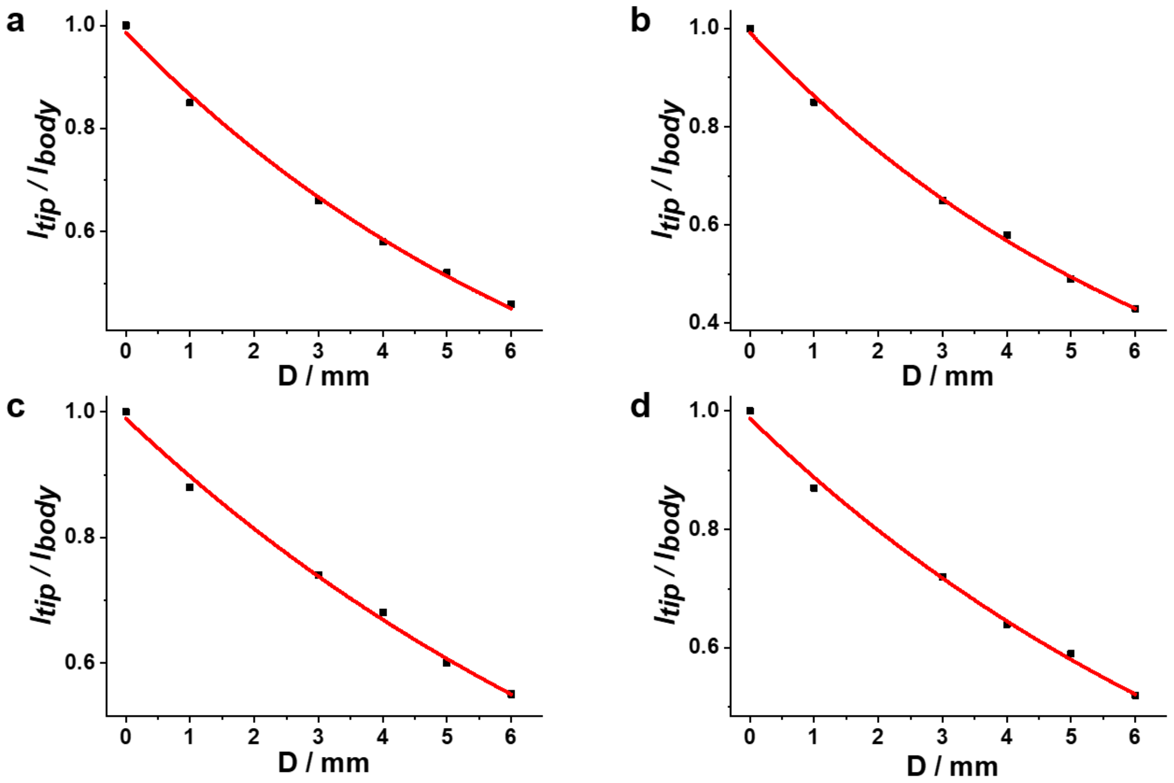

Supplementary Figure 22. Decay of intensity with distance, I_{tip}/I_{body}. (a) uncoated straight state, **2**, (b) coated straight state, MNP//**2**, (c) uncoated straight state, **3**, and (d) coated straight state, MNP//**3**.

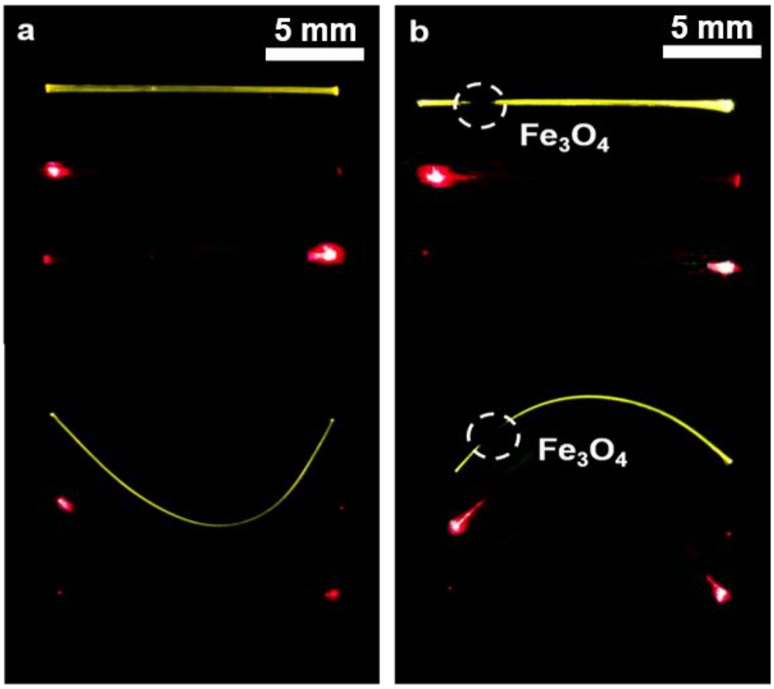

Supplementary Figure 23. Photographs of crystal used as passive optical waveguide. (a) Crystal of **4** in a straight and bent state, (b) MNP//**4** in a straight and bent state.

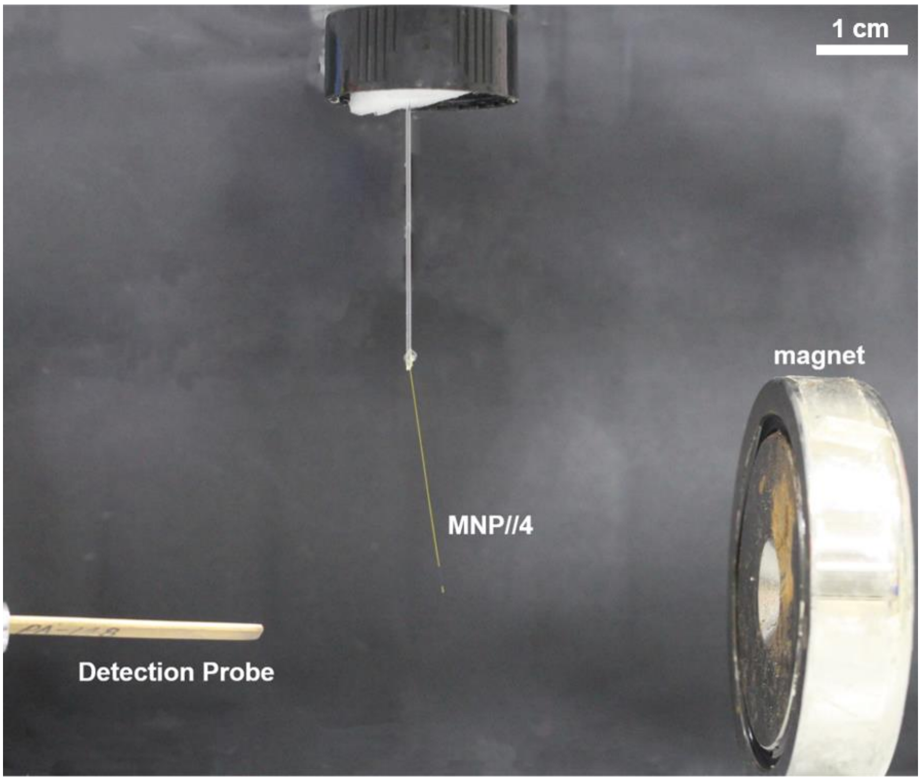

Supplementary Figure 24. Photograph of the experimental setup used for measurement of the magnetic force and crystal bending. Crystals of MNP//**1–6** were first fixed by using glue, and the detection probe of the Gaussmeter was set at the appropriate position where the change of magnetic field (B) can be measured. The crystal was then slowly approached by the magnet.

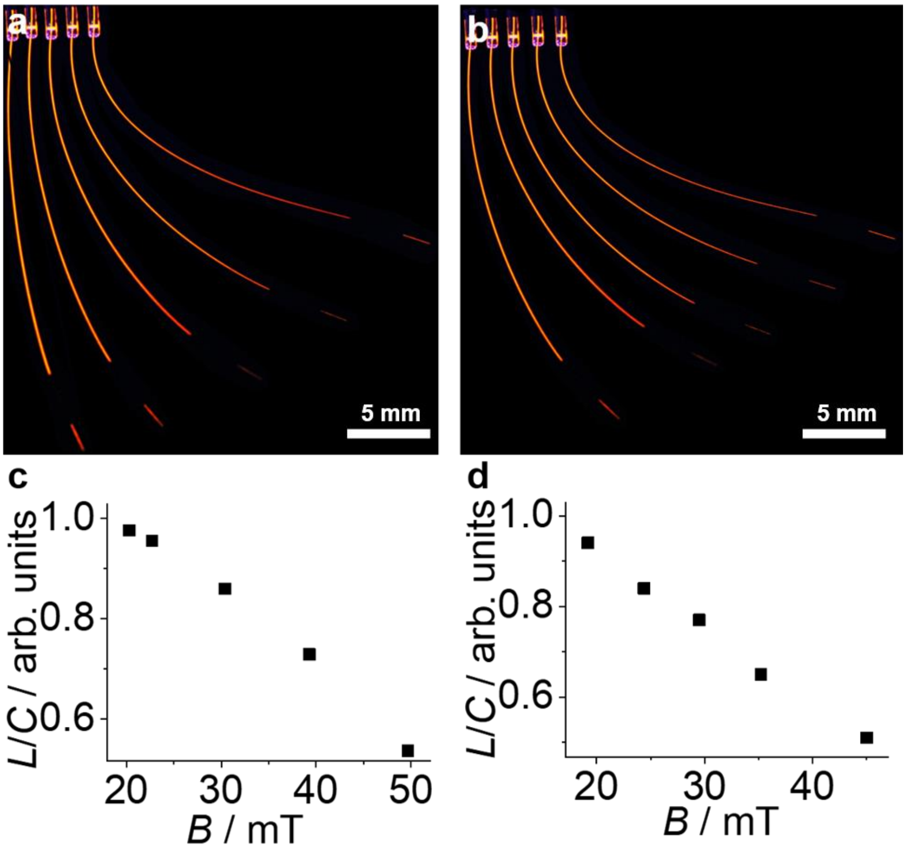

Supplementary Figure 25. Relationship between the magnetic field intensity and crystal bending. (a,b) Images showing bending of MNP//**6** crystals in two tests (a) and (b) while they were being approached with the magnet. (c,d) The ratio of the chord length (*L*) and arc length (*C*) of the crystal plotted against the magnetic field (*B*).

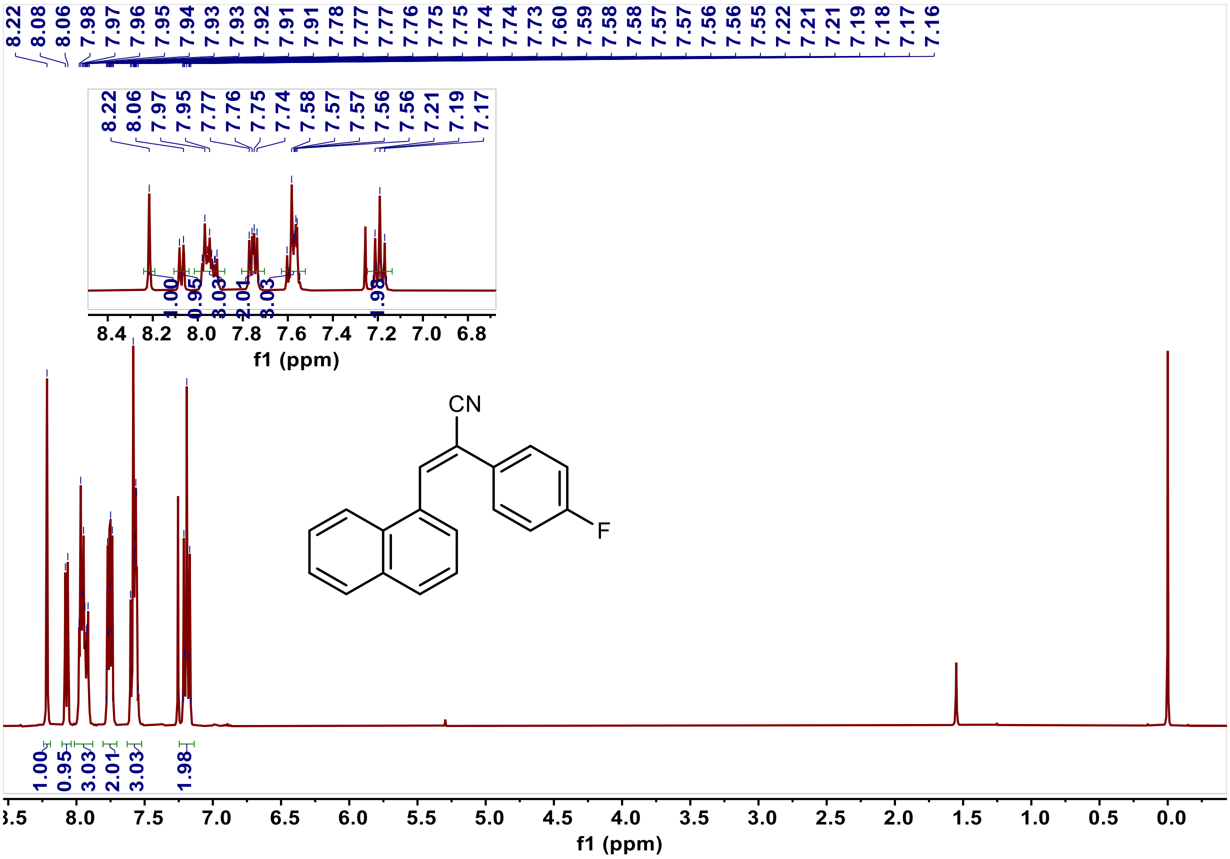

Supplementary Figure 26.^{1}H NMR spectrum of compound **1** (Chloroform-*d*, 400 MHz).

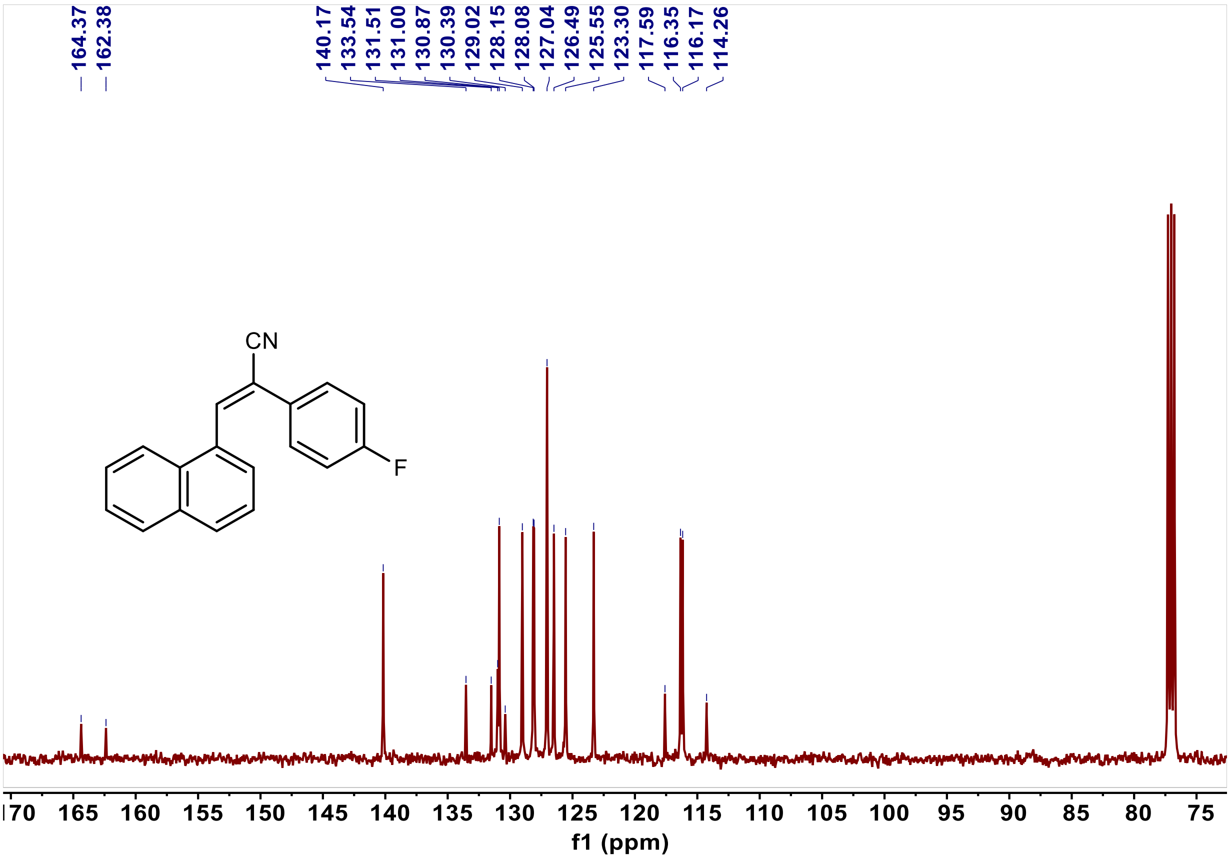

Supplementary Figure 27. ^{13}C{1H} NMR spectrum of compound **1** (Chloroform-*d*, 126 MHz).

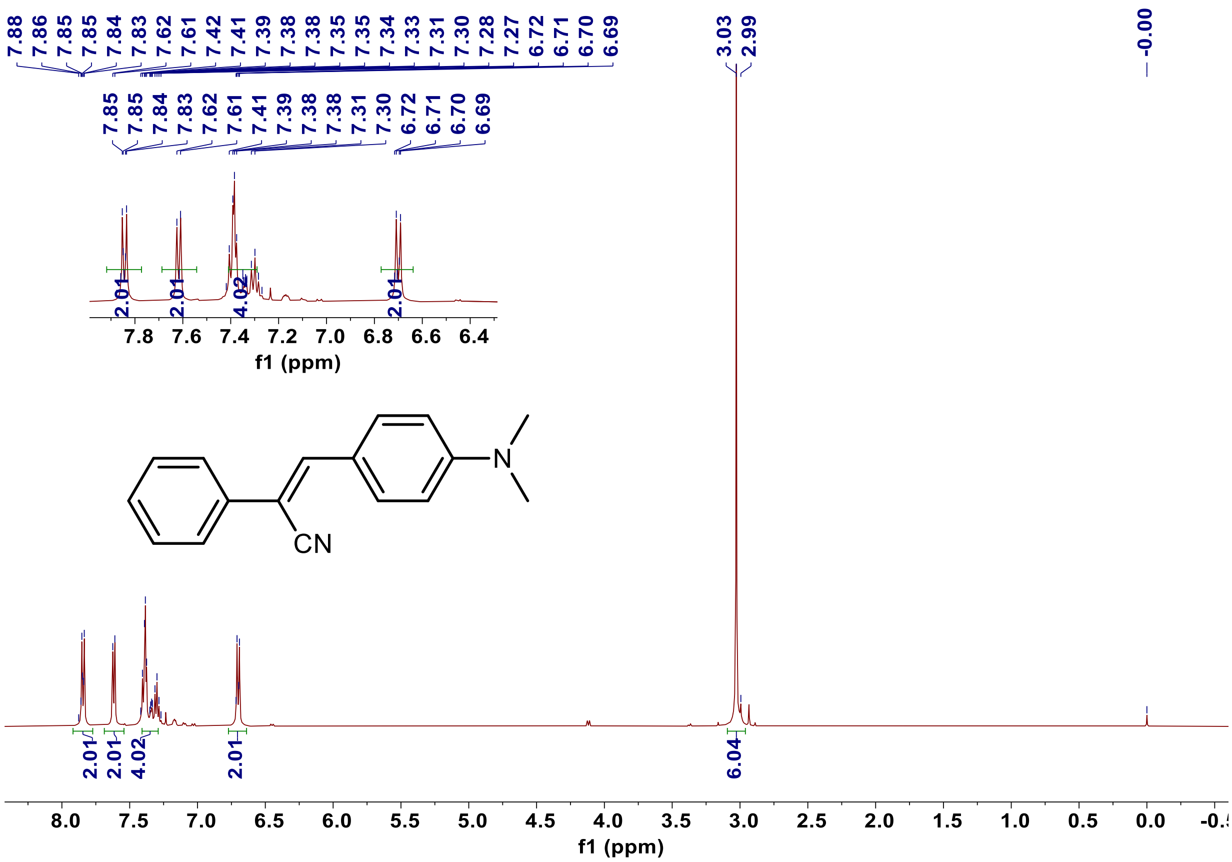

Supplementary Figure 28.^{1}H NMR spectrum of compound **2** (Chloroform-*d*, 500 MHz).

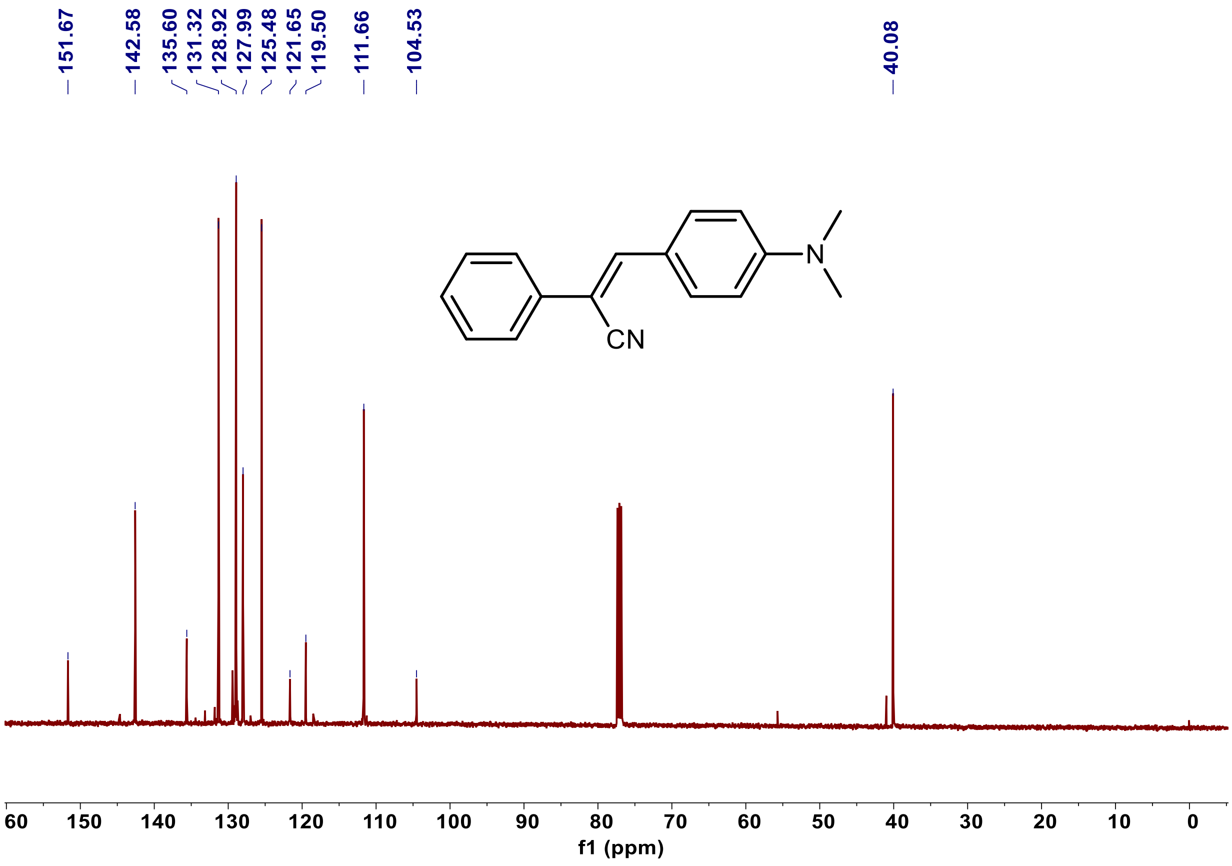

Supplementary Figure 29. ^{13}C{1H} NMR spectrum of compound **2** (Chloroform-*d*, 126 MHz).

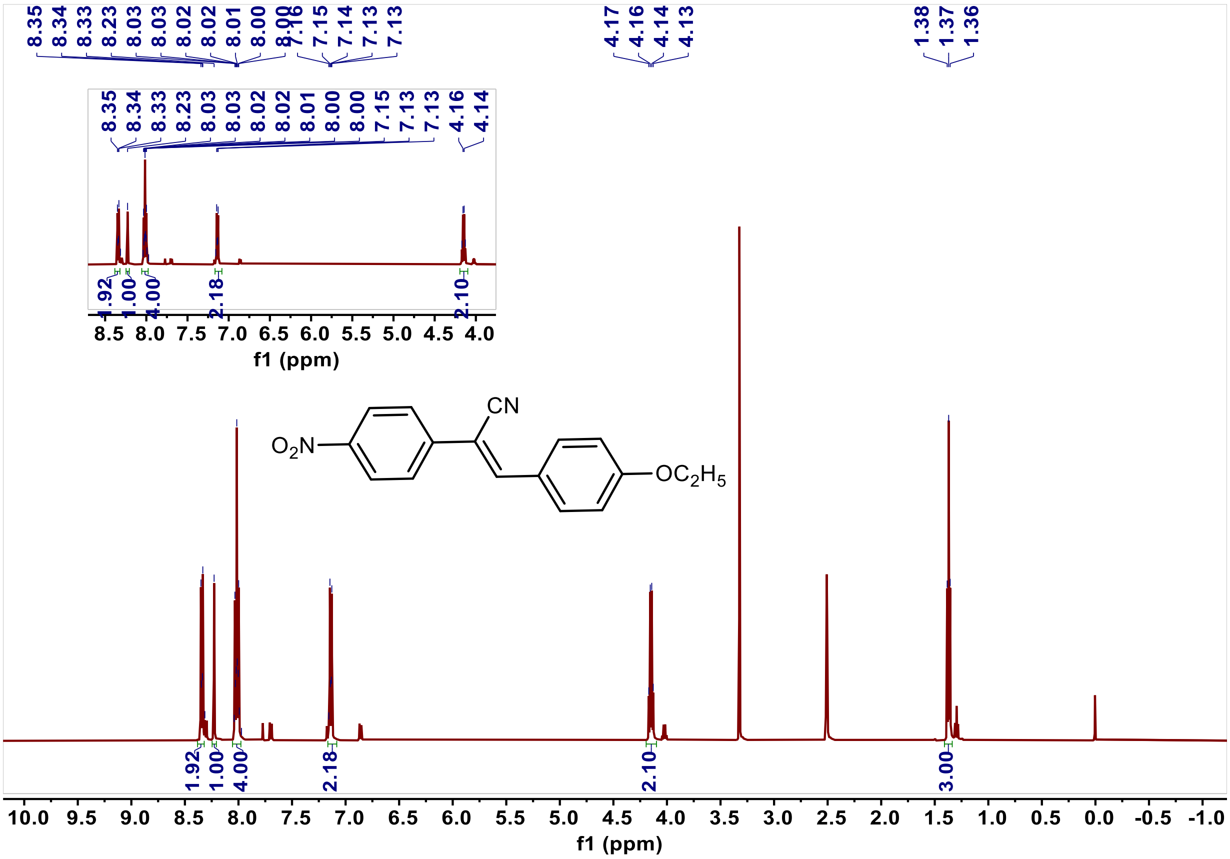

Supplementary Figure 30.^{1}H NMR spectrum of compound **3** (DMSO-d_6, 500 MHz).

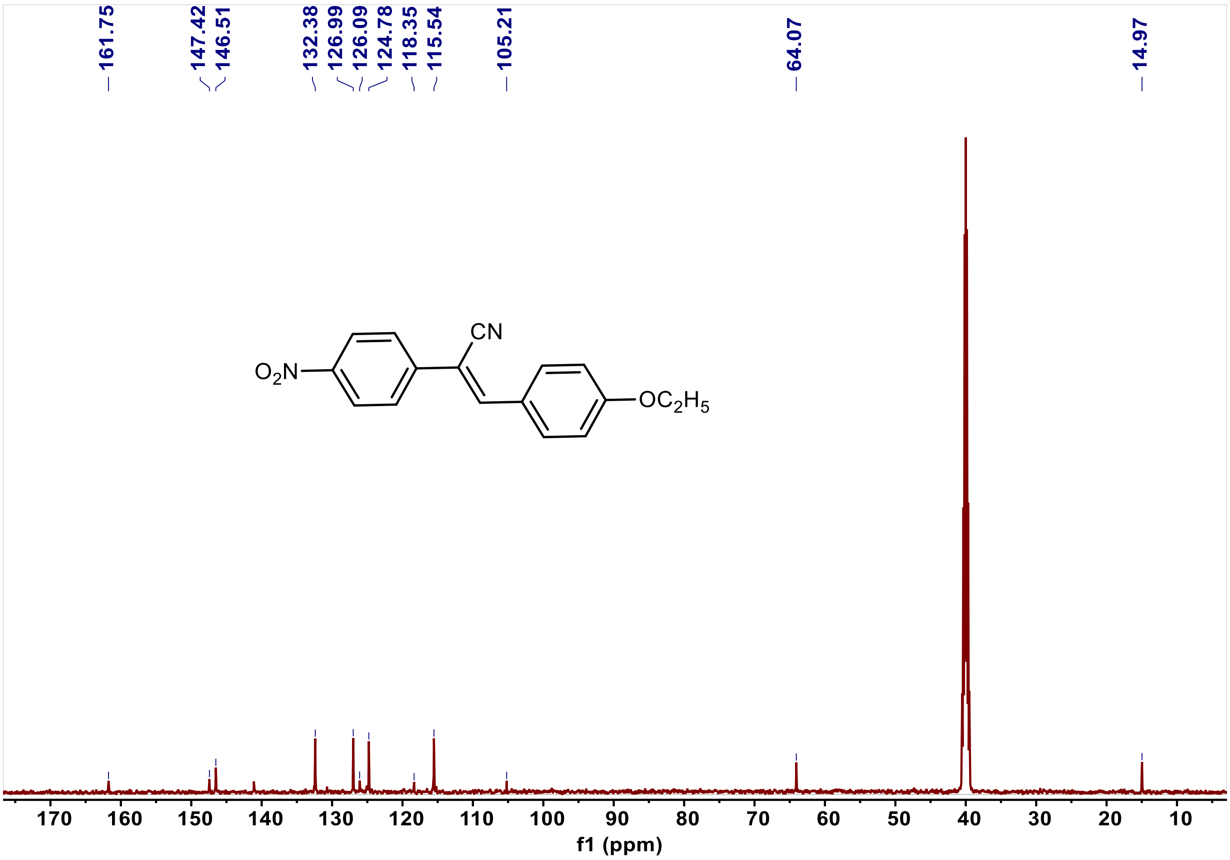

Supplementary Figure 31. ^{13}C{1H} NMR spectrum of compound **3** (DMSO-d_6, 126 MHz).

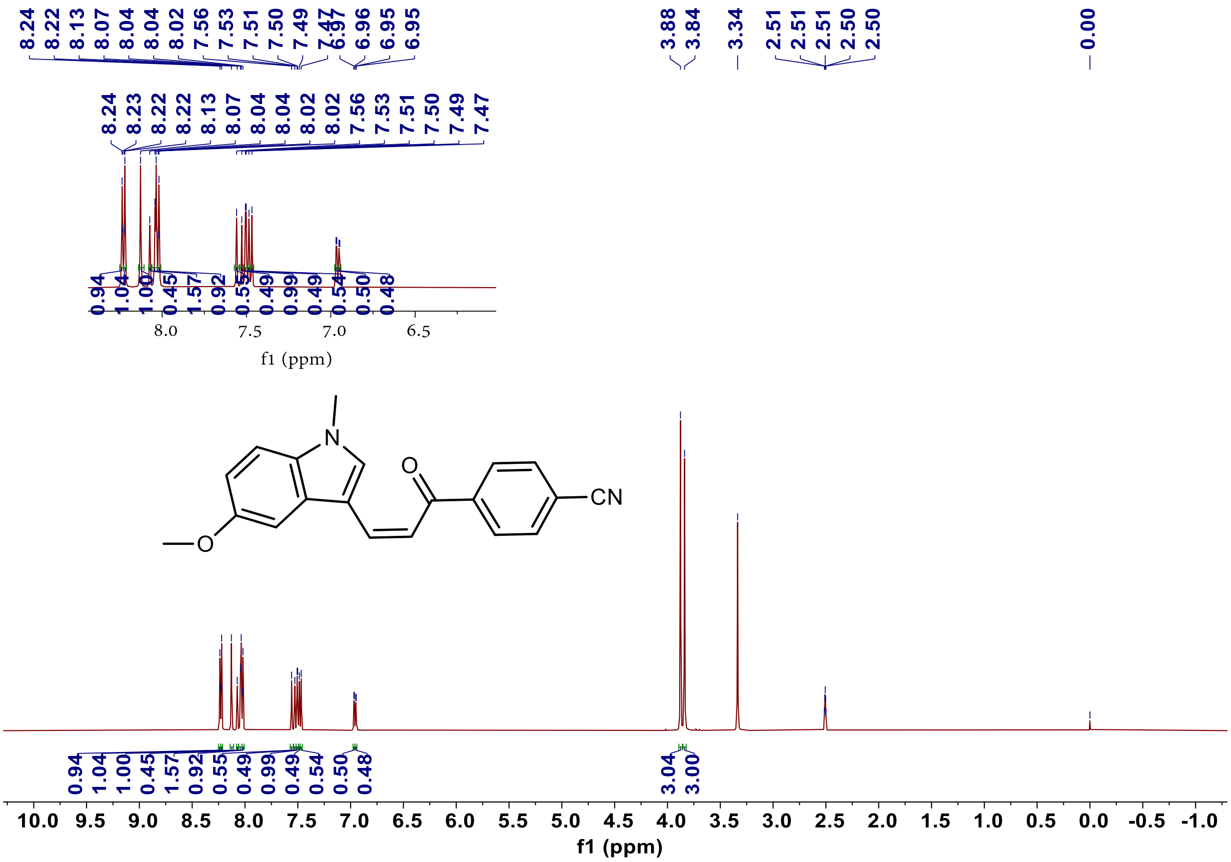

Supplementary Figure 32. ^{1}H NMR spectrum of compound **4** (DMSO-d_6, 500 MHz).

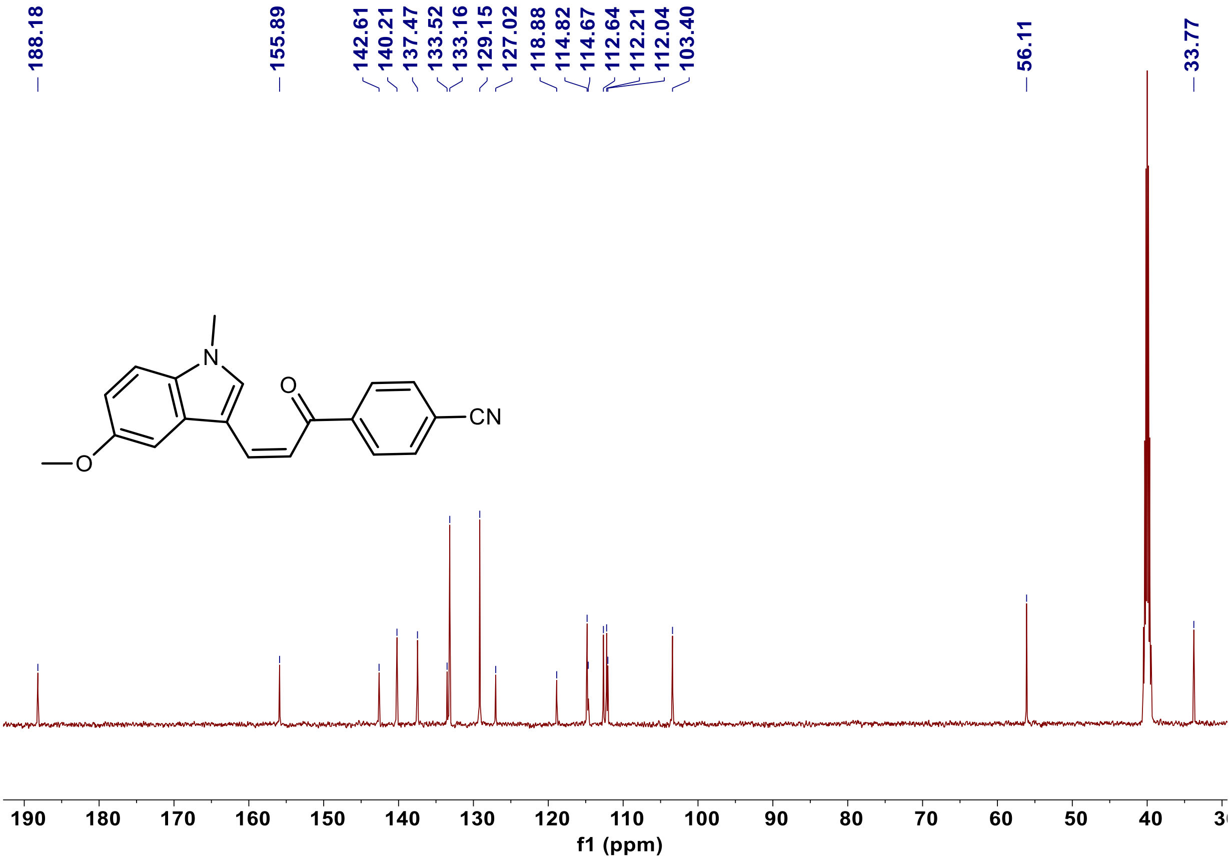

Supplementary Figure 33. ^{13}C{1H} NMR spectrum of compound **4** (DMSO-d_6, 126 MHz).

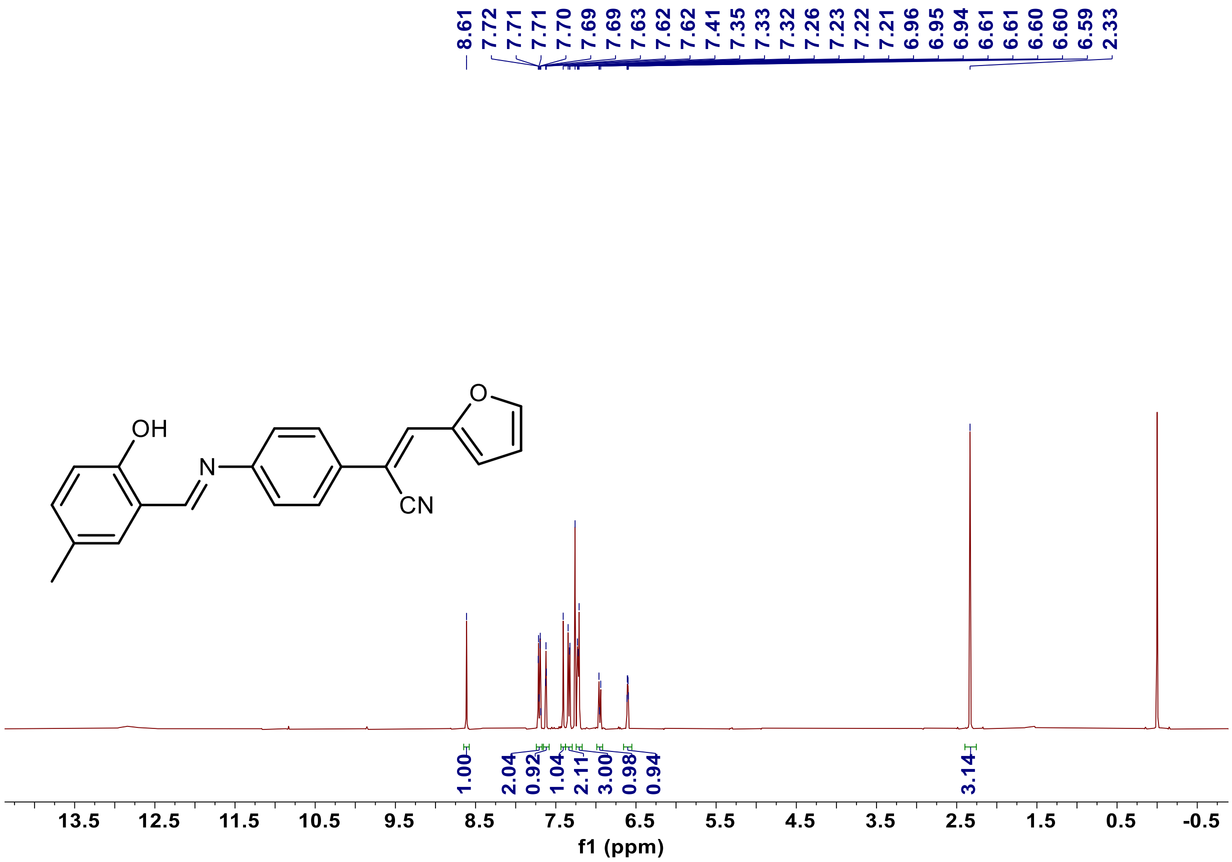

Supplementary Figure 34.^{1}H NMR spectrum of compound **5** (Chloroform-*d*, 400 MHz).

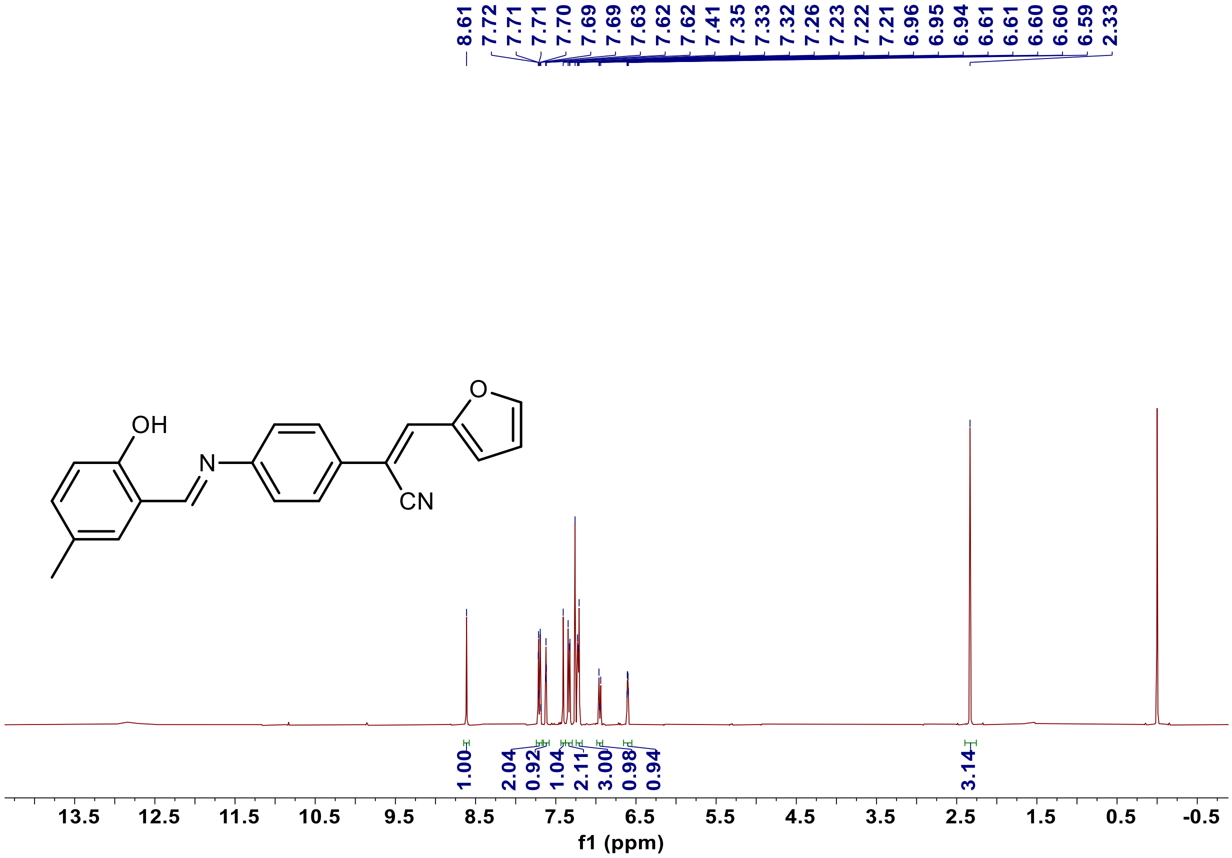

Supplementary Figure 35. ^{13}C{1H} NMR spectrum of compound **5** (DMSO-d_6, 126 MHz).

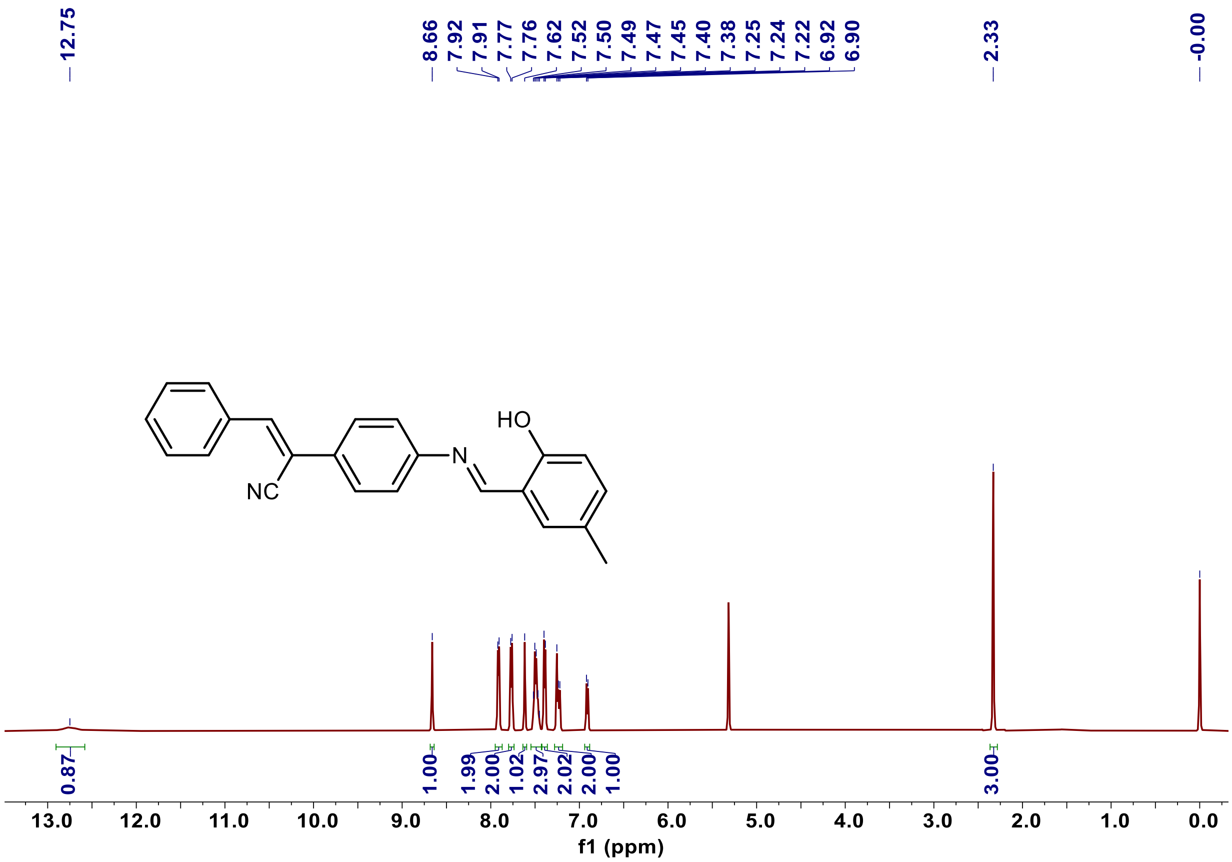

Supplementary Figure 36.^{1}H NMR spectrum of compound **6** (Methylene Chloride-d_2, 500 MHz).

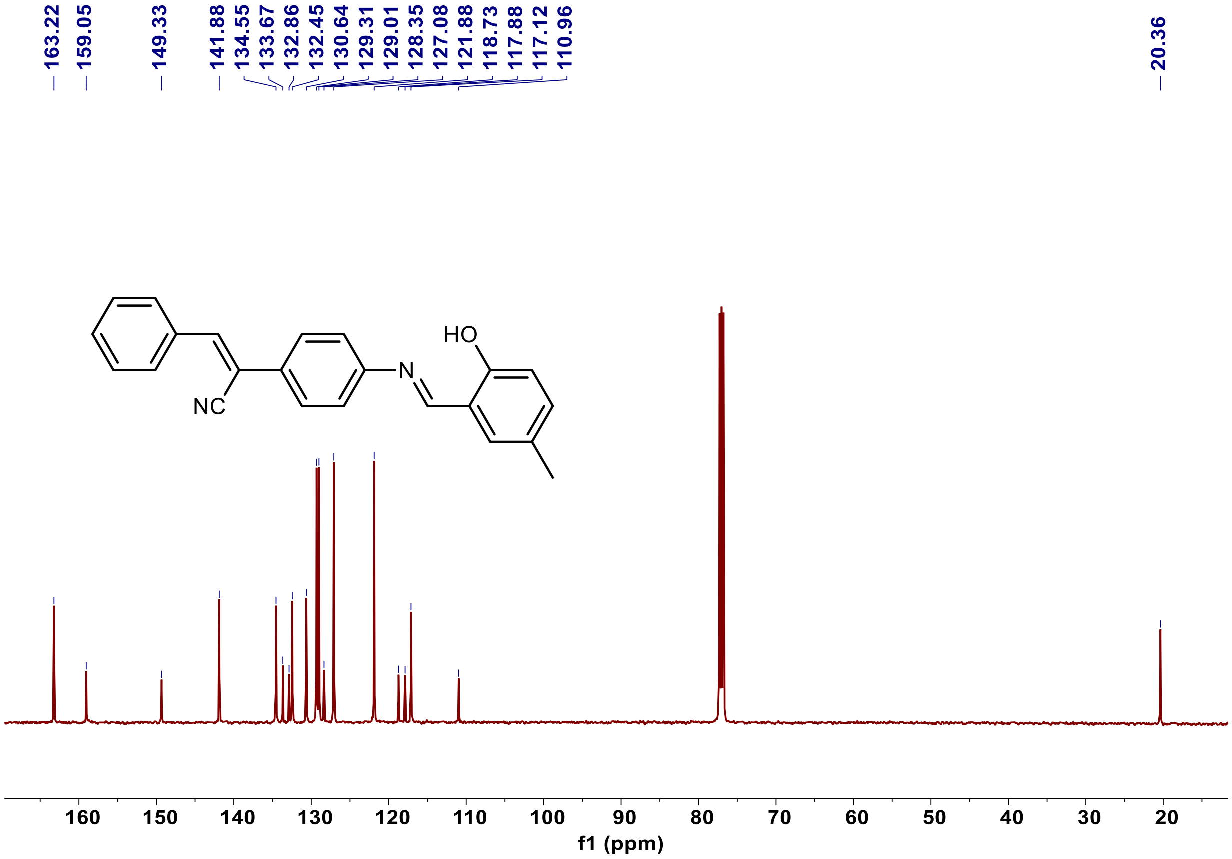

Supplementary Figure 37. ^{13}C{1H} NMR spectrum of compound **6** (Chloroform-*d*, 126 MHz).

Supplementary Tables

Supplementary Table 1. Crystallographic data for crystals of 1 and 3

**Compound**	**1**	**3**
Formula	$C_{19}H_{12}FN$	$C_{17}H_{14}N_2O_3$
Temperature / K	100.0	100.0
Formula Weight	273.30	294.30
Crystal System	orthorhombic	monoclinic
Space Group	$Pna2_1$	*Pn*
a / Å	13.1856(6)	19.656(3)
b / Å	26.1422(11)	3.9147(6)
c / Å	3.8665(2)	19.893(3)
α / °	90	90
β / °	90	106.489(7)
γ / °	90	90
Volume / Å^3	1332.78(11)	1467.8(4)
Z	4	4
Density / (g cm^{-3})	1.362	1.332
μ /mm^{-1}	0.089	0.093
F_{000}	568.0	616.0
Reflections collected	16413	29574
Independent reflections	3542	5173
CCDC deposition No.	2116768	2117194

Supplementary Table 2. Length, width, and thickness of crystals of 1–6

**Compound**	**Length**	**Width**	**Thickness**
**1**	2-3 cm	110-250 μm	65-95 μm
**2**	1-3 cm	100-500 μm	50-200 μm
**3**	1-3 cm	290-450 μm	30-80 μm
**4**	2-4 cm	108-219 μm	40-112 μm
**5**	1-3 cm	160-220 μm	10-22 μm
**6**	2-4 cm	130-150 μm	20-35 μm
